# How Microsolvation Affects the Balance of Atomic Level Mechanism in Substitution and Elimination Reactions: Insights into the Role of Solvent Molecules in Inducing Mechanistic Transitions

**DOI:** 10.3390/molecules30030496

**Published:** 2025-01-23

**Authors:** Gang Fu, Hongyi Wang, Wenqing Zhen, Xin Zhou, Li Yang, Jiaxu Zhang

**Affiliations:** 1 MIIT Key Laboratory of Critical Materials Technology for New Energy Conversion and Storage, School of Chemistry and Chemical Engineering, State Key Laboratory of Urban Water Resource and Environment, Harbin Institute of Technology, Harbin 150001, China; 20b925088@stu.hit.edu.cn (G.F.); wanghongyi999123@163.com (H.W.); 20b925102@stu.hit.edu.cn (W.Z.); zhoux@hit.edu.cn (X.Z.); 2 Key Laboratory of Chemistry and Chemical Engineering on Heavy-Carbon Resources, School of Chemistry and Chemical Engineering, Yili Normal University, Yining 835000, China

**Keywords:** S_N_2/E2 reaction, microsolvation effect, direct dynamics simulation, atomic level mechanism

## Abstract

Solvents play a crucial role in ion–molecule reactions and have been used to control the outcome effectively. However, little is known about how solvent molecules affect atomic-level mechanisms. Therefore, we executed direct dynamics simulations of the HO^−^(H_2_O_w_) + CH_3_CH_2_Br system to elucidate the dynamics behavior of chemical reactions in a microsolvated environment and compared them with previous gas-phase data. Our results show that the presence of a single water solvent molecule significantly suppresses the direct mechanism, reducing its ratio from 0.62 to 0.18, thereby promoting the indirect mechanism. Spatial effects and prolonged ion–molecule collisions combine to drive this mechanism shift. Among them, water molecules impede the reactive collisions of HO^−^ and CH_3_CH_2_Br, while at the same time, the attractive interaction of hydrogen bonds between ions and molecules produces long-lived intermediates that favor the indirect mechanism. On the other hand, microsolvation also affects the reaction preference of the S_N_2 and E2 channels, which is more conducive to stabilizing the transition state of the S_N_2 channel due to the difference in solute–solvent interactions, thus increasing the competitiveness of this pathway. These results emphasize the profound influence of solvent molecules in regulating reaction selectivity and underlying microscopic mechanisms in more complex systems.

## 1. Introduction

Bimolecular nucleophilic substitution (S_N_2) and base-induced elimination (E2) occupy a unique place in organic chemistry due to their apparent simplicity as well as utility, and they usually co-exist and compete [1]. Intensive experimental [2,3,4,5,6,7,8,9,10,11] and theoretical [12,13,14,15,16,17,18,19,20,21] investigation of S_N_2/E2 reactions over several decades has provided valuable insights into unraveling the origins of the competing mechanism. For instance, Bickelhaupt and Ren et al., in their study of the model reaction X^−^ + CH_3_CH_2_Y, observed systematic periodic trends in the S_N_2 and E2 reactions. They further revealed that electronegativity, basicity, and steric effects influence the energy barriers of S_N_2/E2 processes, thereby altering the reaction preferences [22,23,24]. However, a clear distinction between S_N_2 and E2 reactions is a difficult challenge because several aspects have similar effects on the competing reactions: nucleophile/base attack [25,26], leaving group [17], reaction temperature [27], and solvent properties [18,28,29]. Among them, the solvent’s dielectric constant, steric effects, and dragging phenomena control the progression of ion–molecule reactions in solution by altering the geometry and electronic properties of the molecules [30,31,32]. The specifics of reactions occurring in solution are typically deduced rather than directly observed because the trajectories of molecules are heavily influenced by frequent collisions with a substantial volume of solvent [29]. This interaction obscures the ability to discern and evaluate the dynamic behavior of microscopic reaction mechanisms. Consequently, the concept of microsolvation, involving a small number of solvent molecules surrounding reactant ions, has attracted considerable attention as a transitional framework linking the gas and liquid phases [31,33]. It offers a bottom-up perspective for investigating solute–solvent interactions, thereby enhancing our comprehension of hydration reaction dynamics.

The ability to add solvent molecules one by one in the vicinity of the reactant ions has been widely used to study how the reaction is affected [34,35,36]. The velocity distribution of the product ions in the plane of scattering in the HO^−^(H_2_O)_n=0–2_ + CH_3_I reaction shows significant differences in scattering patterns when the HO^−^ reactant is bound with one or two water molecules [31]. The results show that solvent water inhibits the forward-scattered products, while the HO^−^(H_2_O) cluster directs the reaction predominantly toward the backward-scattered I^−^ products through steric guidance. Only isotropic scattering and lower product velocities were observed with further addition of water molecules. Xie et al. reproduced the experimental results of the HO^−^(H_2_O)_n_ + CH_3_I reaction by direct dynamics simulations and also found that the S_N_2 and proton transfer (PT) pathways in the gas phase were equally important [37,38,39]. However, adding water molecules to the reaction inhibited the PT pathway, allowing the S_N_2 reaction to dominate. Therefore, water molecules can control the ratio of microscopic mechanisms in chemical reactions through spatial effects and influence the selectivity between different reactions. The presence of solvated water also produces interesting dynamic behaviors that open additional reaction channels [18,35,40,41,42]. For example, the HOO^−^(H_2_O) + CH_3_Y (Y = Cl, I) system undergoes frequent proton transfer in the deep potential well at the entrance channel of the potential energy surface (PES), where the monohydrated peroxide ion behaves as a shapeshifting nucleophile [35,36]. Thus, nucleophilic reagents with different nucleophilic centers can be generated, contributing to the emergence of novel pathways. The steric effect of solvent molecules is shown in the F^−^(H_2_O) + CH_3_I reaction to exhibit different interactions, with dragging being more critical than shielding during nucleophilic substitution. Further, individual solvent molecules significantly inhibited the reactivity of F^−^ and iodomethane due to the combined dynamical effect of interaction time, steric hindrance, and collision-induced dehydration [30]. This provides a new perspective on understanding how microsolvation affects reactivity. The researchers have extensively explored S_N_2 reactions in other protic/aprotic solvent environments, except for investigating the influence of aqueous molecules on S_N_2 reactions. The results of HOO^−^(NH_3_)_n_ + CH_3_Cl show that NH_3_ as solvent molecules similarly increases the reaction energy barrier for S_N_2 reactions, and there is a good linearity between the reaction energy barriers and the transition state geometrical looseness and asymmetry, as well as charge asymmetry [41]. Wu et al. further investigated the impact of microsolvation on the S_N_2 reaction and extended it to the E2 reaction [18]. Zhang’s group investigated how methanol molecules affect the competition between S_N_2 and E2 reactions [28]. The results show that the addition of a single methanol solvent molecule leads to notably different dynamical behaviors, which mainly favor the S_N_2 reaction. This effect is attributed to the distinct solute–solvent interactions at the central barrier, which stabilize the transition state for substitution. However, the exploration of the effects of microsolvation on atomic dynamics is still in its early stages. Additionally, when hydrogen atoms near the α-carbon are replaced with alkyl chains, the E2 pathway opens and competes with the S_N_2 reaction, potentially leading to more complex and varied reaction dynamics [43,44].

HO^−^, as an isoelectron of F^−^, exhibits markedly different dynamic behavior in reactions, including unexpected reaction pathways [45] and a reversal of dominant atomistic mechanisms [38,46,47,48,49,50]. Previous studies have shown that under low collision energy conditions, the F^−^ + CH_3_CH_2_Y (Y = Cl, Br) reactions primarily proceed via an indirect mechanism, with solvent molecules further enhancing this trend [13,28]. In contrast, the HO^−^ + CH_3_CH_2_Y (Y = Cl, Br) reactions are dominated by the direct mechanism [50,51]. For systems where S_N_2 and E2 pathways coexist and the direct mechanism prevails, the precise impact of water solvent molecules on reaction selectivity and microscopic mechanisms remains unclear. Furthermore, ion flow tube experiments [6] indicate that the reaction rate constant of HO^−^(H_2_O_w_) + CH_3_CH_2_Br is approximately twice that of HO^−^(H_2_O_w_) + CH_3_CH_2_Cl, and thus the former requires fewer trajectories to be simulated when experimental accuracy is achieved. To control computational costs and explore the origin behind the issues, we chose the HO^−^(H_2_O_w_) + CH_3_CH_2_Br reaction for direct dynamics simulation to reveal the role played by the microsolvation effect in the transformation of the atomic level mechanism. In addition, we also calculated the electronic structure of HO^−^(H_2_O_w_)_n=0–3_ + CH_3_CH_2_Br system and attempted to elucidate how the stepwise solvation affects the reaction energy barrier through a two-step energy decomposition analysis (EDA). Direct dynamics simulations and electronic structure calculations shed light on the reasons for the change in reaction channel preferences as well as the shift in atomic-level mechanisms as the degree of solvation increases.

## 2. Results and Discussions

### 2.1. Potential Energy Surface

We calculated the PES for the HO^−^(H_2_O_w_) + CH_3_CH_2_Br reaction using the B3LYP/ECP/d method, as shown in Figures S1–S3, and the detailed geometry is listed in Figure S4. Because previous studies have shown that solvated nucleophilic reagents can induce the generation of new shapeshifting nucleophilic reagents [35], we have also calculated the PES for the reaction of O_w_ as a nucleophilic center with CH_3_CH_2_Br, as shown in Figure S5, and the corresponding geometrical parameters are listed in Figure S6. The calculations show that the PES of the nucleophilic reagents HO^−^(H_2_O_w_) and shapeshifting nucleophilic HO_w_^−^(H_2_O) have the same shape and relative energies. Therefore, we speculate that nucleophilic reagents with different nucleophilic centers may have similar dynamical behaviors, in which the shapeshifting nucleophilic reagents should have equivalent competitiveness in the reaction. Since HO^−^(H_2_O_w_) and HO_w_^−^(H_2_O) have the same PES, only the HO^−^(H_2_O_w_)_n_ with CH_3_CH_2_Br reaction is considered in the subsequent calculations for the solvated systems. As shown in Figures S7–S10, we further calculated the stable point properties of HO^−^(H_2_O_w_)_2–3_ + CH_3_CH_2_Br reactions and compared them with the gas phase HO^−^ + CH_3_CH_2_Br reaction in our previous study to explore the effect of stepwise solvation on S_N_2/E2 selectivity and the microreaction mechanism [51]. In addition, we calculated the PES of the HO^−^ + CH_3_CH_2_Br reaction under the implicit solvation model to examine how the liquid phase environment affects the chemical reaction, and the relative energies and geometrical parameters of the stabilization points are listed in Figures S11 and S12. Through analysis, we found that the number of water molecules has an essential effect on the energy of the stabilization point of the PES, so we focused on exploring how the reaction channel changes as the degree of solvation increases.

#### 2.1.1. Effect of Solvation on Potential Energy Surface

**Figure 1:**
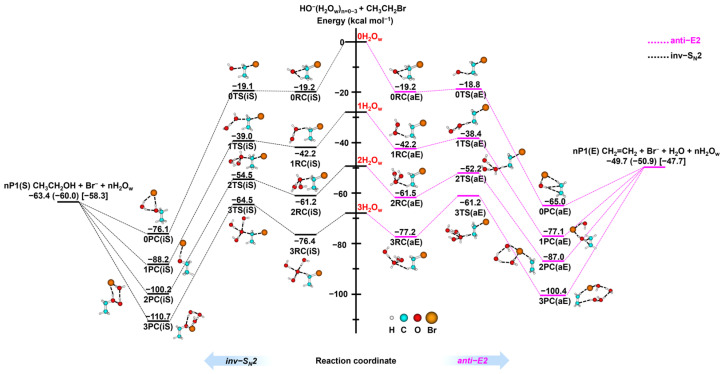
Schematic PES for the HO^−^(H_2_O_w_)_n=0−3_ + CH_3_CH_2_Br reactions illustrate the stationary points along the inv-S_N_2 (depicted in black) and anti-E2 (highlighted in pink) pathways, as calculated at the B3LYP/ECP/d level of theory. The energy values, expressed in kcal mol^−1^, are referenced to the isolated reactants without incorporating zero-point energy (ZPE). Values in parentheses include ZPE corrections, while experimental data, presented in square brackets, are derived from the Active Thermochemical Tables (ATcT) [52].

The two most crucial reaction channels in the HO^−^(H_2_O_w_)_n=0–3_ + CH_3_CH_2_Br reaction, namely the inv-S_N_2 and anti-E2 reactions, are demonstrated as shown in Figure 1. For the gas phase reaction, detailed data can be obtained from our previous studies [51], while the complete potential energy profiles and geometric parameters under microsolvation conditions are shown in Figures S1–S10. To facilitate the discussion, we list the relative energy values of S_N_2, E2, and PT pathway stabilization points calculated under microsolvation and polarizable continuum model (PCM) in Table 1. Previous results have shown that for the gas phase HO^−^ + CH_3_CH_2_Br reaction, inv-S_N_2 is the energy minimizing pathway, followed by the anti-E2 reaction and that the similarity of the transition state energy barriers of the two means that they compete with each other in the reaction. Due to spatial resistance effects, the ret-S_N_2 and syn-E2 channels do not compete with the significant pathway. Except for the S_N_2 and E2 reactions, the transition state energy barriers for the proton transfer (PT) reaction are even lower than those for the syn-E2 reaction. However, producing heat-absorbing products implies that this channel cannot occur at room temperature. Surprisingly, all five reaction pathways are connected via the same entrance channel ion–dipole complex, and studies have shown that this intermediate configuration facilitates the direct mechanism.

With the introduction of a single solvent water molecule, the minimum energy pathway for the HO^−^(H_2_O_w_) + CH_3_CH_2_Br reaction remains the inv-S_N_2 channel as shown in Figure 1. The nucleophilic reagent HO^−^(H_2_O_w_) comes close to CH_3_CH_2_Br to form pre-reactive complexes 1RC (iS), and after attacking α-C from the backside crosses 1TS (iS) to form the post-reaction complex 1PC (iS), which ultimately yields the substitution product 1P1 (S) CH_3_CH_2_OH + Br^−^ + H_2_O_w_. The nucleophilic reagent can also attack α-C from the frontside, forming 1P1 (S) after crossing the high energy barriers 1TS (rS), as shown in Table 1. If the base abstracts β-H, the formation of the pre-reaction complex 1RC is followed by the arrival of the post-reaction complex 1PC via the 1TS (aE) or 1TS (sE) transition state, which ultimately produces the elimination product 1P1 (E) CH_2_=CH_2_ + Br^−^ + H_2_O + H_2_O_w_. The presence of water molecules opens up additional channels for solvation products, and multiple solvation products may be formed in addition to the independent reaction products 1P1 (S) and 1P1 (E), such as 1P2 (S)~1P4 (S) and 1P2 (E)~1P10 (E) in Figure S3, where the solvation products appear to be more competitive than the independent products due to lower energy. For the PT channel, adding water molecules increases the energy barrier significantly, which becomes less competitive than the syn-E2 channel. Based on the central energy barrier, we propose prioritizing different reaction channels as inv-S_N_2 ~ anti-E2 > syn-E2 > PT > ret-S_N_2. In addition, the water molecules also change the geometry of the complexes at the entrance channel of the PES, and due to the steric effect of the water molecules, the monohydration reaction involves not only ionic dipole pre-reactive complexes (1RC (sE), 1RC (rS)), but also hydrogen-bonded pre-reactive complexes (1RC (aE), 1RC (iS), and 1RC (PT)), which are close to each other in terms of energy, as shown in Table 1. The water molecule lifts the energy of the potential energy surface as a whole in addition to affecting the structure of the stabilization point, which implies a decrease in the reactivity of the HO^−^(H_2_O_w_) + CH_3_CH_2_Br system. Moreover, the nucleophilic HO^−^(H_2_O_w_) can generate the shapeshifting nucleophilic reagent HO_w_^−^(H_2_O) with new nucleophilic centers through proton exchange, which reacts with CH_3_CH_2_Br with the same potential energy profile and geometry. Therefore, how nucleophilic reagents with different nucleophilic centers affect the reaction dynamics is an interesting question that deserves further exploration.

When two water molecules are added to the HO^−^ + CH_3_CH_2_Br system, Table 1 shows that the energy value of the stationary point of the PES changes considerably. Only the inv-S_N_2 and anti-E2 channels were submerged entirely below the reactant energy zero point, and their product thermal energy release was 0.3 and 14.0 kcal mol^−1^, respectively, thus thermodynamically favoring the substitution reaction pathway. In addition to ret-S_N_2, the total energy barrier of syn-E2 is 4.4 kcal mol^−1^, which is also above the reactant energy zero. At the same time, the transition state of the PT pathway disappeared, and the nucleophilic reagent HO^−^(H_2_O_w_)_2_ could directly extract α-H and absorb the energy to produce the product 2P1 (PT). When the degree of solvation is further increased, the shape of the PES is similar to that of the double hydration systems. However, the increase in water molecules further increases the energies of all stable points of the PES, with all channels having energies above the asymptote of the zero point of the energy of the reactants, as shown in Table 1. Therefore, if the microdynamics processes in the HO^−^(H_2_O_w_)_3_ + CH_3_CH_2_Br system are studied, a high initial collision energy must be given. On the other hand, the study of the HO^−^ + CH_3_CH_2_Br system in implicit solvents allows for the exploration of the reaction in the liquid phase environment, and Figure S11 shows that the PES in the liquid phase environment is quite different from those in the gas phase and microsolvent environments, with a transition from a double well PES to a single peak profile.

#### 2.1.2. Energy Decomposition Analysis

**Figure 2:**
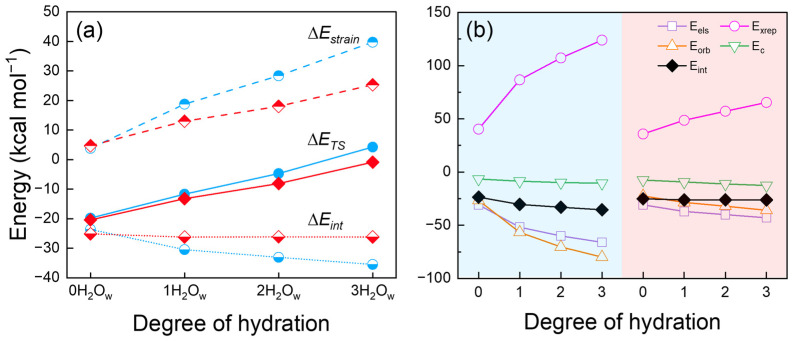
(**a**) Energy decomposition analysis (EDA) performed at the B3LYP-D3/ECP/d level for the TS of the microsolvated HO^−^(H_2_O_w_)_n=0–3_ + CH_3_CH_2_Br reactions, comparing the inv-S_N_2 (red line) and anti-E2 (blue line) pathways. (**b**) Results of the sobEDA analysis at the same theoretical level for the TS of the microsolvated HO^−^(H_2_O_w_)_n=0–3_ + CH_3_CH_2_Br reactions, presented with a red background for inv-S_N_2 and a blue background for anti-E2. Where ∆*E*_int_: interaction energy, ∆*E*_els_: electrostatic energy, ∆*E*_x_: exchange energy, ∆*E*_rep_: Pauli repulsion, ∆*E*_orb_: orbital interaction, ∆*E*_DFTc_: correlation energy, ∆*E*_dc_: dispersion correction. Among them, ∆*E*_int_ = ∆*E*_els_ + ∆*E*_xrep_ + ∆*E*_orb_ + ∆*E*_c_, ∆*E*_xrep_ = ∆*E*_x_ + ∆*E*_rep_, ∆E_c_ = ∆*E*_DFTc_ + ∆*E*_dc_.

The energy decomposition of the transition state of the S_N_2/E2 channel with varying degrees of solvation indicates that the rise in the overall energy barrier is predominantly attributable to the geometric distortion of the reactants throughout the reaction (as illustrated in Table S1). Among these, the structural distortion of CH_3_CH_2_Br from the reactant to the transition state is the primary contributor to the increased strain energy. This conclusion is further substantiated by the root mean square deviation (RMSD) between the structures of the reactant (CH_3_CH_2_Br) and the corresponding transition state fragments, as illustrated in Figure S13. As the number of water molecules increases from zero to three, the RMSD of the inv-S_N_2 channel increases from 0.05 to 0.17, while the RMSD of the anti-E2 channel increases from 0.07 to 0.19. Figure 2a further illustrates that the overall barriers to the inv-S_N_2 and anti-E2 channels rise for different reasons as the degree of solvation increases. The inv-S_N_2 channel exhibits little variation in interaction energy, with most of the observed increase in the overall energy barrier attributed to the rise in strain energy resulting from enhanced solvation. In contrast, in the anti-E2 channel, the reduction in the interaction energy (*E_int_*) between the reactants is inadequate to counterbalance the higher strain energy (*E_strain_*) resulting from geometric distortion as the degree of solvation increases, leading to the general increase in the overall energy barrier. In summary, the transition state geometry of the anti-E2 channel is more susceptible to alterations in the degree of solvation, resulting in a more pronounced rise in its energy barrier. Consequently, as the degree of solvation increases, the energy barrier difference between the inv-S_N_2 and anti-E2 channels gradually widens.

A more detailed energy decomposition analysis was conducted to gain further insight into the varying trends in interaction energy between the inv-S_N_2 and anti-E2 channels as the number of water molecules changes. Figure 2b and Table S2 demonstrate that in the anti-E2 channel, an increase in the degree of solvation results in a proportional increase in the contribution of orbital interactions (*E_orb_*) to the interaction energy, which subsequently exceeds that of electrostatic effects (*E_els_*). The proportion of the total attractive effect increases from 31% to 35% to 37% to 38%, while the proportion of electrostatic effects decreases from 36% to 32% to 31% to 31%. The dispersion effect (*E_c_*) constituted the most negligible contribution. Furthermore, the exchange-repulsion effect (*E_xrep_*) substantially negatively influenced the overall interaction energy, counteracting 63% to 77% of the total attractive force. However, the downtrend trends of orbital interactions and electrostatic effects still outweighed the energy instability caused by the exchange-repulsion effect, resulting in a gradual decrease in the total interaction energy. In contrast, in the inv-S_N_2 channel, electrostatic effects consistently represent the largest contributor to the total attractive interaction. In conjunction with orbital interactions and a small dispersion term, they effectively counteract the effects of exchange-repulsion during solvation, thereby maintaining the interaction energy as relatively constant. These findings elucidate the reason why inv-S_N_2 and anti-E2 channel interaction energy can show different trends with increasing solvation.

### 2.2. Reaction Dynamics Simulations

By following the atomic motion along the reaction pathway through chemical dynamics trajectory simulations, we can advance beyond merely predicting the reaction mechanism based on stationary points. Therefore, we performed trajectory simulations of the HO^−^(H_2_O_w_) + CH_3_CH_2_Br reaction using the B3LYP/ECP/d methodology and compared them with our previous dynamic results for the gas-phase HO^−^ + CH_3_CH_2_Br reaction to elucidate the effect of stepwise solvation on the reaction selectivity and the microscopic reaction mechanism [51].

#### 2.2.1. Reactivity

**Figure 3:**
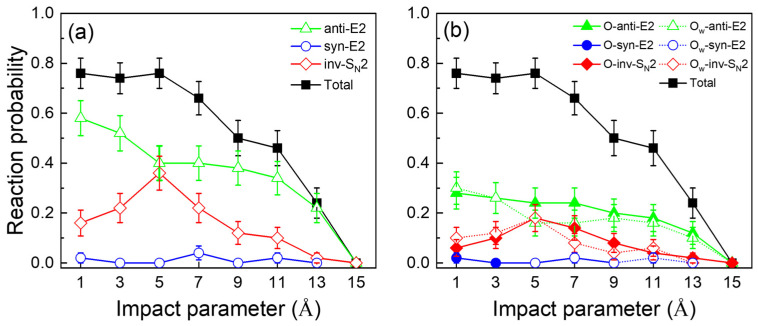
(**a**) Opacity functions *P_r_*(*b*) for the anti-E2, syn-E2, inv-S_N_2, and total reaction channels of HO^−^(H_2_O_w_) + CH_3_CH_2_Br at 300 K. (**b**) Opacity functions *P_r_*(*b*) for the O_w_-anti-E2, O-anti-E2, O_w_-syn-E2, O-syn-E2, O_w_-inv-S_N_2, O-inv-S_N_2, and total reaction channels of HO^−^(H_2_O_w_) + CH_3_CH_2_Br at 300 K.

At a low collision energy of 0.04 eV, three reaction channels are observed in the HO^−^(H_2_O_w_) + CH_3_CH_2_Br systems: anti-E2, syn-E2, and inv-S_N_2. Figure 3a illustrates the reaction probability, *P_r_*(*b*), as a function of the impact parameter, *b*. In the microsolvation reaction, the anti-E2 and inv-S_N_2 pathways exhibit identical *b_max_* values (15 Å) yet display markedly disparate reactivity trends. In the case of the anti-E2 reaction, the *P_r_*(*b*) undergoes a pronounced decline within the range of impact parameters *b* = 1 to 5 Å. However, it exhibits a relatively high probability level at larger impact parameters. When the impact parameter exceeds 11 Å, the probability of the reaction decreases rapidly and reaches zero at 15 Å. In contrast, the *P_r_*(*b*) for the inv-S_N_2 pathway reaches a maximum of 5 Å and gradually decreases to zero. This trend discrepancy suggests a competitive interplay between the S_N_2 and E2 channels, wherein the rise in the inv-S_N_2 channel’s reaction probability exerts an inhibitory effect on anti-E2. The reaction probability of the elimination pathway is consistently higher than that of the substitution pathway across the entire impact parameter range. This phenomenon is because the anti-E2 transition state has a looser geometry, leading to more reactivity at larger impact parameters [12,13]. The syn-E2 channel probability is independent of the impact parameter and does not compete with the two main pathways mentioned above due to the significant steric hindrance caused by the syn-coplanar conformation, which inhibits the reaction. Microsolvated bases can produce shapeshifting nucleophiles with varying nucleophilic centers, and prior research has demonstrated that the reactions involved in shapeshifting nucleophilic reagents are not competitive [35,36,41,42]. However, our findings indicate that the O_w_- and O-channels exhibit comparable reaction possibilities, as shown in Figure 3b. which can be attributed to the frequent proton transfer in the entrance channel of the potential energy surface and the relatively shallow transition energy barrier (0.09 kcal mol^−1^) between HO^−^(H_2_O_w_) and HO_w_^−^(H_2_O).

We can obtain the integral cross sections (ICSs, σ) using $\int_{0}^{b_{max}}P_{r}\left(b\right)2\pi bdb$ based on the opacity functions. In HO^−^(H_2_O_w_) + CH_3_CH_2_Br reaction, the σ of anti-E2, syn-E2, inv-S_N_2, and total pathways are 180.2 ± 9.59, 6.2 ± 1.5, 78.7 ± 6.1, and 265.1 ± 22.8 Å^2^, respectively. The results of the ICSs showed that the order of the three channels is anti-E2 > inv-S_N_2 > syn-E2, implying that anti-E2 was still the dominant reaction in the solvated system. The reaction rate constant for the reactants at a 300 K thermal energy, i.e., *E_coll_* = 0.04 eV, is given by *k*(*E_coll_*, *T_v_*, *T_r_*) = *v*(*E_coll_*) × *σ*(*E_coll_*, *T_v_*, *T_r_*), where *v*(*E_coll_*) is the relative velocity of the reactants, *T_v_*/*T_r_* is the vibrational/rotational temperature of CH_3_CH_2_Br at 300 K, and *σ* is the total reaction cross section for dynamics simulations. Based on the equation, the *k* obtained for the target reaction is (1.41 ± 0.12) × 10^−9^ cm^3^ molecule^−1^ s^−1^. It agrees with the experimental rate (1.7 × 10^−9^ cm^3^ molecule^−1^ s^−1^) measured using ion flow tube experiments under the same conditions [6]. We also compared the reaction with solvent-free HO^−^ + CH_3_CH_2_Br. The theoretical and experimental rate constants are (1.77 ± 0.07) × 10^−9^ and 2.6 × 10^−9^ cm^3^ molecule^−1^ s^−1^, respectively [6]. The results show that solvent-free reactions are about 1.5 times more reactive than single-hydration reactions. This reactivity suppression phenomenon caused by the solvent is also found for the X^−^(sol) + RY(X = F; Y = Cl, Br, I; R = Me, Et) reactions [28,30,53,54,55].

The solvation of bases opens up new reaction pathways and produces solvation products. For convenience of discussion, the oxygen atom in the nucleophile and the oxygen atom in the water are no longer distinguished when classifying the products. In the trajectory simulation of the HO^−^(H_2_O) + CH_3_CH_2_Br reaction, six E2 product channels (1P1 (E), 1P3 (E)~1P5 (E), 1P9 (E), 1P10 (E)) and four S_N_2 channels (1P1 (S) to 1P4 (S)) are observed, whose energetic features are indicated in Figure S3. For the E2 and S_N_2 reactions, it is thermodynamically favorable to generate solvated products. However, the simulation results show that the solvated products are significantly inhibited in terms of dynamics compared to the isolated products, similar to the hydrated S_N_2 reaction [56]. The contribution ratios of the microsolvated S_N_2 and E2 products to the target reactions are 6.3% and 10.4%, respectively, while the generation channels of the isolated products 1P1(S) and 1P1(E) dominate, accounting for 24.3% and 59.0% of the substitution and elimination events, respectively. This phenomenon can be attributed to subtle intramolecular vibrational energy redistribution (IVR) [28]. When the reaction crosses the transition state, releasing a large amount of potential energy can cause the shearing of solvent molecules, ultimately leading to the decomposition of the complex and the formation of isolated products. In the trajectories of desolvated S_N_2 and E2 products, about 79% and 85% of the trajectories follow the complete fragmentation pathway, ultimately producing high-energy products 1P1(S) (CH_3_CH_2_OH + Br^−^ + H_2_O) and 1P1(E) (CH_2_=CH_2_ + Br^−^ + 2H_2_O).

Further statistics show that the bare Br^−^ and Br^−^(sol) ratio is 0.91:0.09. Most of the bare Br^−^ comes from the fully dissociated channels 1P1(E) and 1P1(S), which contribute 59.0% and 24.3%, respectively. The remaining small amount of Br^−^ comes from channels 1P3(E), 1P5(E), 1P9(E), and 1P3(S) with contributions of 0.4%, 5.9%, 0.6%, and 1.0%, respectively. In contrast, Br^−^(sol) comes mainly from channels 1P4(E), 1P10(E), 1P2(S), and 1P4(S) with contributions of 2.6%, 0.9%, 2.6%, and 2.7%, respectively, but accounts for a tiny fraction of the dynamics. These results suggest that in the microsolvation system, the products are more likely to form high-energy isolated species rather than the more energetically favorable solvation products.

#### 2.2.2. Atomic-Level Mechanism

**Figure 4:**
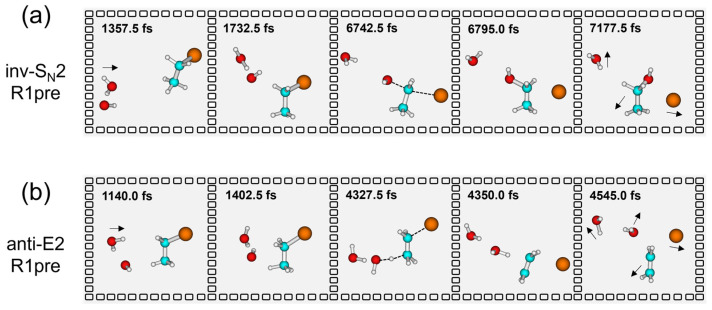
Atomistic snapshots of representative trajectories highlighting the dominant pathways: (**a**) inv-S_N_2 and (**b**) anti-E2 in the HO^−^(H_2_O_w_) + CH_3_CH_2_Br reaction. The reaction follows an indirect pathway, involving the formation of a 1RC complex (R1pre) within the reactant entrance potential energy well (refer to Figure 1).

Dynamics simulations provide insights into the intricate atomic motions associated with different reaction mechanisms. In the HO^−^(H_2_O_w_) + CH_3_CH_2_Br system, both substitution and elimination reactions occur through a combination of direct and indirect pathways (refer to Animations S1–S8 in the Supplementary Materials). In contrast to the gas phase HO^−^ + CH_3_CH_2_Br reaction [51], water molecules inhibit the direct mechanism, facilitating the indirect mechanism, in which pre-mechanism is vital. For example, Figure 4a shows a snapshot of the trajectory of the pre-mechanism in the inv-S_N_2 pathway of the single hydrated system, where first the nucleophilic reagent HO^−^(H_2_O_w_) approaches CH_3_CH_2_Br and is then trapped by the entrance channel potential well trap, crossing the Walden inversion transition state after interaction of about 5000 fs, followed by rapid segregation into nucleophilic substitution products. Figure 4b illustrates the details of atomic motions in the anti-E2 pathway, where the nucleophilic reagent HO^−^(H_2_O_w_) is captured by the deep potential well after a collision with CH_3_CH_2_Br at ~1402 fs, forming a long-lived intermediate of ~2925 fs. Then, the base extracted β-H atom crosses the transition state of the anti-E2, followed by a fast segregation into the elimination product.

**Figure 5:**
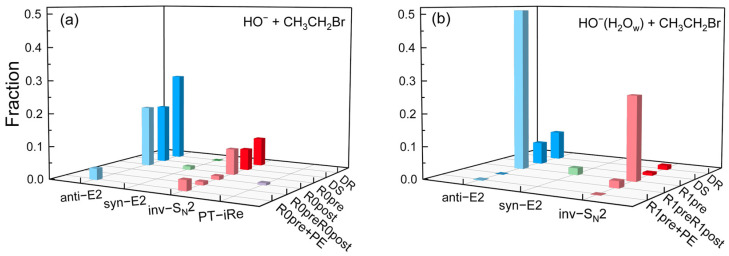
Branching ratios of atomistic mechanisms for (**a**) HO^−^ + CH_3_CH_2_Br involving S_N_2, E2 and PT-iRe channels at 300 K [51] and (**b**) HO^−^(H_2_O_w_) + CH_3_CH_2_Br involving S_N_2 and E2 channels at 300 K. Dark-colored bars indicate direct reaction pathways, specifically rebound (DR) and stripping (DS) mechanisms, while lighter-colored bars represent indirect processes. Indirect mechanisms involve the formation of pre-reaction complexes—0RC (R0Pre) or 1RC (R1Pre)—and post-reaction complexes—0PC(E)/0PC(iS) (R0Post) or 1PC(E)/1PC(iS) (R1Post)—as well as the proton exchange mechanism (PE)—which dynamically couples these events.

The mechanistic branching ratios for the HO^−^(H_2_O_w_)_n=0–1_ + CH_3_CH_2_Br reactions, as shown in Figure 5, demonstrate the essential difference between the dynamics of the solvent-free and solvated reactions. Overall, the anti-E2 channel dominates both HO^−^(H_2_O_w_)_n=0–1_ + CH_3_CH_2_Br reactions, with anti-E2, syn-E2, inv-S_N_2, and PT-iRe yields of 68.7%, 1.2%, 29.4%, and 0.7% for HO^−^ [51] and 67.1%, 2.2%, 30.7%, and 0.0% for HO^−^(H_2_O_w_). The addition of water molecules slightly inhibited the branching ratio of the anti-E2 reaction, thereby promoting the inv-S_N_2 reaction. The mechanistic contributions of direct and indirect events in the anti-E2, syn-E2, inv-S_N_2, and PT-iRe channels are 0.46:0.23, 0.00:0.01, 0.16:0.13, and 0.00:0.01 at *n* = 0 and the branching ratios when *n* = 1 are 0.16:0.51, 0.00:0.02, 0.02:0.29, and 0.00:0.00, respectively. The presence of water molecules increased the indirect mechanism in the anti-E2 channel from 0.23 to 0.51. Similarly, the proportion of the indirect mechanism in the inv-S_N_2 reaction rose by 16%. These findings indicate that water molecules suppress the direct mechanism in the primary pathway, thereby facilitating the indirect mechanism.

**Figure 6:**
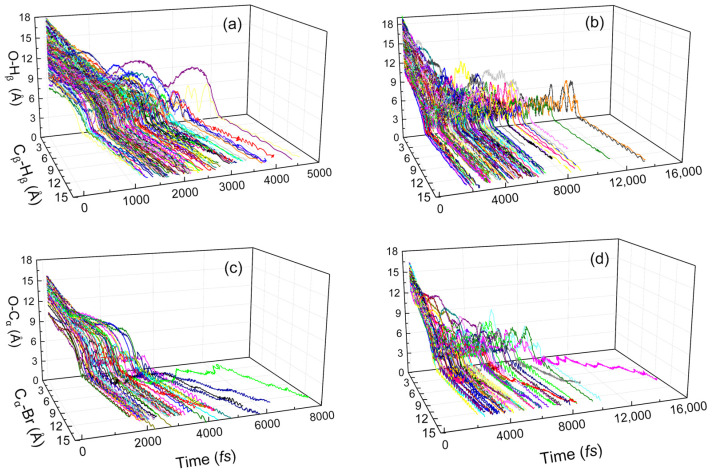
Time evolution of key bond lengths during the reaction. (**a**) anti-E2 in HO^−^ + CH_3_CH_2_Br, (**b**) anti-E2 in HO^−^(H_2_O_w_) + CH_3_CH_2_Br, (**c**) inv-S_N_2 in HO^−^ + CH_3_CH_2_Br, and (**d**) inv-S_N_2 in HO^−^(H_2_O_w_) + CH_3_CH_2_Br.

To investigate why the microsolvation leads to the direct mechanism to indirect mechanism shift, we counted the key bond lengths of all reaction trajectories concerning time, as shown in Figure 6. For the anti-E2 reaction, the reaction duration of the solvent-free system is within 0~5000 fs, as demonstrated in Figure 6a, while the presence of water molecules prolongs the reaction duration, most of the trajectories are distributed in the interval of 0~10,000 fs, as shown in Figure 6b. By comparing the solvent-free and solvated systems, we found that the nucleophilic reagent of the solvent-free system can directly attack β-H to have a direct reaction, and the O-H_β_ keeps decreasing and finally reaches a constant value as shown in Figure 6a. However, for the solvated system, the nucleophilic reagent cannot directly attack β-H due to the obstruction of water molecules, which leads to the O-H_β_ bond fluctuating within a specific range for a long time, as shown in Figure 6b. Until the intermolecular vibrational mode conforms to the reaction’s vibrational direction, the elimination reaction will occur across the transition state. Similarly, adding water molecules inhibits the direct mechanism in the inv-S_N_2 channel, prompting the reaction trajectory duration to change from a 0~6000 fs distribution to a 0~10,000 fs distribution. This phenomenon can be attributed to the significantly higher frequency of O-C_α_ bond length fluctuations in the solvated system compared to the solvent-free system, as illustrated in Figure 6c,d. In conclusion, the participation of water molecules prevents the direct reaction collision between CH_3_CH_2_Br and the nucleophilic reagent, and only after pushing away the water molecules can the nucleophilic reagent attack the α-C/β-H to react, which fundamentally enhances the indirect mechanism. We further counted the interaction time from the first point of collision of the reaction to the transition state, as shown in Figure S14. The interaction time of solvent-free reactions is mainly distributed in the range of 0 to 2 ps, and more than half of the trajectories react immediately upon collision. The presence of water molecules hinders reactive collisions, which must pass through long-lived intermediate complexes to react. Therefore, the interaction time of a single hydration reaction is widely distributed between 0 and 12 ps. The weak coupling between low-frequency intermolecular modes and high-frequency intramolecular modes constitutes a dynamics bottleneck for transferring the energy necessary to reach the E2 or S_N_2 transition states, which leads to the trapping of the ion–molecule complexes at the entrance channel of the potential energy surface [28]. Consequently, direct events are strongly suppressed upon solvation, and the indirect trajectories control the elimination and substitution dynamics. This result can also be supported by the wave function analysis of the intermediates at the entrance channel of the potential energy surface, as shown in Figure S15. Both the IGMH and the electrostatic potential indicate that with the addition of water molecules, hydrogen bonding interactions replace a portion of the ion–dipole interactions, contributing to the structural deviation of the pre-reactive complexes from the transition state, and hence the need to adjust the conformation to the proper orientation before reactive collisions occur. This is the reason for the prevalence of indirect mechanisms.

**Figure 7:**
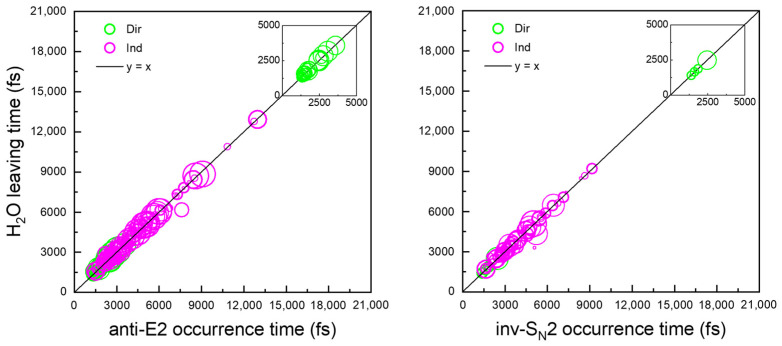
Distribution of the time for solvating water to depart as a function of the occurrence time of the E2 or S_N_2 reactions for the HO^−^(H_2_O) + CH_3_CH_2_Br system. The departure time of water is determined when the HOH···O/Br bond distance reaches approximately 2.2 Å, the upper limit for a typical hydrogen bond, while the occurrence time of the E2 or S_N_2 reaction is recorded when the reactive system crosses the E2 (O–H_β_ bond distance ≤ 1 Å) or S_N_2 (C_α_–O bond distance ≤ 2 Å) transition state. The results are presented for the individual reaction mechanisms: Direct (green circle) and Indirect (pink circle). Data for Dir are also shown in the inset for clarity.

The statistical model for the HO^−^(H_2_O) + CH_3_CH_2_Br reaction with equilibrium microsolvation posits that the water molecule remains in interaction with the reactive system throughout the S_N_2 or E2 reaction, leading to the formation of water-solvated products. These solvated products may eventually dissociate if they possess enough energy. However, the dynamics of water solvation observed in the trajectories exhibit a distinctly different behavior from that predicted by the model, providing insight into why the reaction tends to favor the formation of isolated products. This discrepancy is illustrated by comparing the time for water to separate from the reaction system (i.e., HOH···O/Br breaking bond ≥ 2.2 Å) [57] with the time required for the base-induced elimination or substitution to occur. Figure 7 presents this information as a scatter plot, showing the prominent anti-E2 and inv-S_N_2 pathways with similar characteristics. For trajectories leading to the formation of bare ionic products, the departure of H_2_O during the S_N_2 reaction (C_α_–O bond distance ≤ 2 Å) coincides with the displacement of Br^−^ by HO^−^, while in the E2 reaction, the abstraction of β-H (HO–H_β_ bond distance ≤ 1 Å) occurs simultaneously. In both cases, water departs at the moment when elimination or substitution takes place. This behavior represents the preferred hydrodynamics for multiple product channels, accounting for 94% of the solvation reactions observed.

Simulations also reveal that in the majority of indirect trajectories (more than 94%), H_2_O remains attached to HO^−^ during the early stages of interaction with CH_3_CH_2_Br. As the reaction progresses, it enters the 1RC pre-reaction potential energy well and transitions to the product asymptote via either the E2 transition state 1TS (aE)/1TS (sE) or the S_N_2 transition state 1TS (iS). Once the system passes through the transition state, overcoming the dynamical bottleneck, and forms the C_α_-OH or HO–H_β_ bond, the hydrogen bonding between H_2_O and HO (HOH···OH) becomes weaker. The energy released into the H···OH stretch mode tends to break the hydrogen bond, facilitating the detachment of water. Consequently, H_2_O solvates the transition state but does not remain with the products.

#### 2.2.3. E2 Versus S_N_2 Competition and Solvation Effects

**Figure 8:**
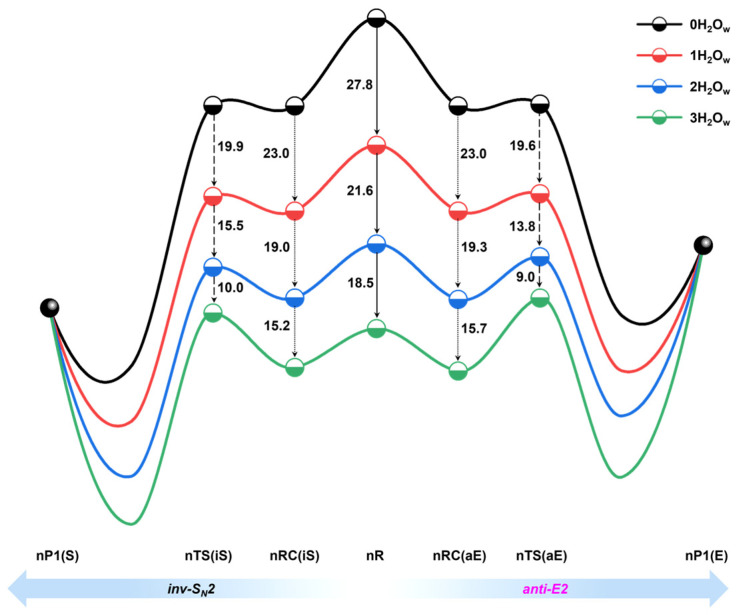
Schematic diagram of the extent of water solvent stabilization of the stable point of the anti-E2 and inv-S_N_2 channels in the reaction HO^−^(H_2_O_w_)_n=0–3_ + CH_3_CH_2_Br.

An energy decomposition analysis reveals different origins of the rise in energy barriers for the inv-S_N_2 and anti-E2 channels under microsolvation. As the degree of solvation increases, the interaction energy in the inv-S_N_2 channel remains almost constant, and the increase in the energy barrier is attributed solely to the strain energy. Conversely, in the anti-E2 channel, interaction energy decreases with increasing solvation, but this reduction is insufficient to offset the rise in strain energy, resulting in an increase in the energy barrier. In addition, the energy barrier difference between inv-S_N_2 and anti-E2 gradually increases with the increase in the number of water molecules, and the dynamics results also indicate that adding a single water molecule increases the branching ratio slightly of the inv-S_N_2 channel. To elucidate how the presence of water molecules raises the reaction energy barrier and thus inhibits the reactivity and how it increases the competitiveness of the inv-S_N_2 reaction, we simplified the potential energy curve as shown in Figure 8. The results show that the gradual addition of water molecules stabilizes the reactants. This stabilizing effect decreases with the increase in the number of water molecules. The energy of the reactants decreases from 27.8, 21.6 to 18.5 kcal mol^−1^, implying that the gradual addition of water molecules to the reaction of HO^−^ + CH_3_CH_2_Br may be saturated, i.e., when a sufficient number of water molecules are added, the reactants’ energy does not in produce a significant decrease. Xie et al. also found that the number of water molecules leads to solution saturation in the HO^−^(H_2_O)_n=0–4_ + CH_3_CH_2_I reaction [18]. We further found that solvent molecules can stabilize the anti-E2 channel pre-reaction complex. However, it is less stabilized than the reactants, with energies decreasing from 23.0, 19.3 to 15.7 kcal mol^−1^. The inv-S_N_2 channel pre-reaction complexes are similar to the anti-E2 channel, with energies decreasing from 23.0, 19.0 to 15.2 kcal mol^−1^. Because the reactivity is closely related to the reaction energy barrier, we examined the degree of stabilization of the transition state by the solvent, and the results show that the solvent molecules are very poorly stabilized for the transition state, with energies decreasing from 19.6, 13.8 to 9.0 kcal mol^−1^ for the anti-E2 channel, which is much lower than that of the water molecules for the reactants. This phenomenon can be explained by the charge distribution, which is concentrated in the reactants and, therefore, easily solubilized by the protonating solvent to produce more stable interactions, whereas the gradual dispersion of the charge when the transition state occurs leads to solvent desolvation and, therefore weakened interactions [41]. A similar trend is shown in the inv-S_N_2 channel, but water molecules stabilize the inv-S_N_2 transition state slightly more than anti-E2. Thus, it is the difference in the degree of solvent stabilization of the reactants and the transition state that leads to an increase in the reactive energy barrier with increasing solvation and thus inhibits the reactivity. In summary, the difference in solvent–solute interactions in the inv-S_N_2 and anti-E2 channels gradually increases the energy barrier difference between the two, thereby increasing the competitiveness of the inv-S_N_2 channel.

## 3. Computational Method

Previous dynamical theoretical study of the solvent-free HO^−^ + CH_3_CH_2_Y (Y = Cl, Br) reaction has shown that the B3LYP method can accurately characterize the PES while obtaining reliable dynamics results with rate constants that agree with experimental values [50,51]. Therefore, in this paper, we continue to use the B3LYP [58] method in conjunction with the ECP/d [59] basis set to calculate the PES and dynamics of the HO^−^(H_2_O_w_) + CH_3_CH_2_Br reaction. To further determine the reliability of B3LYP in microsolvated reactions, we compared the calculated energy values with those of the CCSD(T)/PP/t//MP2/ECP/d method [60,61], details of which are shown in Figure S1. The results of the calculations show that, excluding the unimportant 1TS (rS) and the 1PC (iS), which has a sizeable structural difference, the mean error between the B3LYP and the CCSD(T) method is 0.12 kcal mol^−1^, which suggests that the B3LYP method is in good agreement with those of the high-level CCSD(T) methods for the microsolvation system. In addition, we performed energy decomposition analyses for nTS (aE) and nTS (iS) at different degrees of solvation, and since the decomposed energy terms contain dispersive effects, we re-optimized the structures using the B3LYP-D3 method [62]. The decomposed energies are listed in Tables S1 and S2. The Gaussian 09 quantization program [63] was used for all PES calculations, while the EDA was performed using the sobEDA package [64]. The wave function analyses, such as independent gradient model analysis based on the Hirshfeld partition of molecular density (IGMH) and electrostatic potential (ESP), were performed using the program Multiwfn [65].

The initial parameters for the direct dynamics of the HO^−^(H_2_O_w_) + CH_3_CH_2_Br reaction were set experimentally [6], with the initial collision energy (*E_coll_*) set to 0.04 eV, corresponding to a reaction temperature of 300 K. The vibrational and rotational energies of the reactants were then sampled from the Boltzmann distribution at 300K. Using a quasi-classical sampling method, the zero point energy (ZPE) of each vibrational mode in the reactant was considered to determine the initial coordinates and momentum of the trajectory. Here, the initial orientation of the reactants was randomly distributed. Trajectories were scanned from a specified impact parameter b (1 Å) to the limiting value (*b_max_*) through step size 2 Å. Fifty trajectories were calculated for each value of b, so the total number of trajectories is about 400. All direct dynamics calculations were computed with the VENUS [66] and NWChem [67] coupling packages.

## 4. Conclusions

The electronic structure calculation and dynamics of the S_N_2 and E2 reactions in the HO^−^(H_2_O_w_)_n=0–3_ + CH_3_CH_2_Br system reveal how solvation influences microscopic mechanisms and the competition between pathways. The key findings are summarized as follows:

(i) Dynamical simulations show that S_N_2 and E2 reactions proceed via direct rebound, direct stripping, and indirect mechanisms. A significant shift occurs when transitioning from the gas phase to a single hydration reaction. The dominant mechanism changes from a preference for direct dynamics to one dominated by indirect mechanisms mediated by entrance channel complexes. This shift arises from water molecules hindering direct collisions between nucleophilic reagents and substrates, forming hydrogen-bonded complexes, prolonging ion–molecule interactions, and increasing the prevalence of indirect dynamics.
(ii) Solvation induces structural transformations in nucleophiles. Proton transfer processes at the entrance channel, enabled by small hydrogen migration energy barriers, create shapeshifting nucleophiles HO_w_^−^(H_2_O) with reactivity comparable to original nucleophiles HO^−^(H_2_O_w_).
(iii) The significant energy release in the exit channel drives rapid separation of products, minimizing interaction of the post-reaction complex. This favors bare Br^−^ as the primary product over complexed or solvated Br^−^, despite the latter being energetically favorable.
(iv) In the gas phase, the anti-E2 pathway dominates over inv-S_N_2 with a ratio of 0.69: 0.29, influenced by dynamic effects. Notably, when HO^−^ dissolves only one H_2_O_w_ molecule, subtle changes in solute–solvent interactions near the transition state alter the relative energies of the inv-S_N_2 and anti-E2 pathways, favoring inv-S_N_2. With the gradual addition of water molecules, the barrier gap between inv-S_N_2 and anti-E2 on the potential energy surface widens. Water molecules more strongly stabilize the S_N_2 transition state and correspondingly inhibit the E2 elimination process. The energy decomposition analysis reveals that the rise of the inv-S_N_2 barrier by solvation is due to the increase in strain energy, while the anti-E2 reaction is due to the rise of the energy barrier because the interaction energy cannot compensate for the strain energy. After all, the water molecules are more prone to distorting the structure of the transition state of the anti-E2 reaction, and thus, the inv-S_N_2 reaction becomes more and more critical with the increase in solvation degree.

Future investigations will aim to encompass a broader range of collision energies to unravel the dynamics of larger solvated HO^−^(H_2_O_w_)_n_ clusters and to elucidate the influence of diverse solvent molecules on competing reaction pathways. Ongoing efforts focus on systematically varying initial conditions—including collision energy, impact parameters, and vibrational-rotational quantum states of the reactants—with the ultimate goal of facilitating meaningful comparisons with experimental observations.

## Figures and Tables

**Table 1:** Relative energy of the stationary points for S_N_2, E2, and PT pathways in the HO^−^(H_2_O)_n=1–3_ + CH_3_CH_2_Br and HO^−^(PCM, H_2_O) + CH_3_CH_2_Br reactions.

Species	B3LYP/ECP/d				
	HO_w_^−^(H_2_O)	HO^−^(H_2_O_w_)	HO^−^(H_2_O_w_)_2_	HO^−^(H_2_O_w_)_3_	HO^−^(PCM)
			anti-E2		
nRC (aE)	−14.4	−14.4	−12.1	−9.3	−0.3
nTS (aE)	−10.6	−10.6	−2.8	6.7	6.8
nPC (aE)	−49.3	−49.3	−37.6	−32.5	−38.6
nP1 (E)	−21.9	−21.9	−0.3	18.2	−
			syn-E2		
nRC (sE)	−14.4	−14.4	−11.6	−8.6	−0.1
nTS (sE)	−2.1	−2.1	4.4	11.9	11.7
nPC (sE)	−49.3	−49.3	−37.6	−31.6	−41.7
			inv-S_N_2		
nRC (iS)	−14.4	−14.4	−11.8	−8.5	−0.1
nTS (iS)	−11.2	−11.2	−5.1	3.4	6.6
nPC (iS)	−60.4	−60.4	−50.9	−42.9	−49.4
nP1 (S)	−35.6	−35.6	−14.0	4.5	−49.3
			ret-S_N_2		
nRC (rS)	−14.4	−14.4	−11.8	−8.5	−1.4
nTS (rS)	18.8	18.8	23.9	29.8	30.0
nPC (rS)	−60.1	−60.1	−49.3	−43.9	−52.3
			PT		
nRC (PT)	−14.5	−14.5	/	/	/
nTS (PT)	2.2	2.2	/	/	/
nPC (PT)	1.1	1.1	/	/	/
nP1 (PT)	32.6	32.6	54.2	72.7	25.6

Notes: Energies are presented in kcal mol^−1^, relative to reactants without zero-point energy correction energy (ZPE).

## Data Availability

The data presented in this study are available in Supplementary Materials.

## Supplementary Materials

The following supporting information can be downloaded at: https://www.mdpi.com/article/10.3390/molecules30030496/s1. The main part of the supporting information includes the energy decomposition analysis of the microsolvation reaction, detailed potential energy surfaces and their corresponding geometrical configurations, interaction time, IGMH and electrostatic analyses, and animations of the trajectories.
