# Supplementary material for: How Microsolvation Affects the Balance of Atomic Level Mechanism in Substitution and Elimination Reactions: Insights into the Role of Solvent Molecules in Inducing Mechanistic Transitions
Source: Molecules. 2025 Jan 23;30(3):496. doi: 10.3390/molecules30030496 (PMC11819716; doi:10.3390/molecules30030496)
Supplement: Supplementary file 1 [file molecules-30-00496-s001.zip › Cartesian Coordinates.pdf]

$HO^{-}(H_2O_w) + CH_3CH_2Br$/ $HO_w^{-}(H_2O) + CH_3CH_2Br$ (B3LYP):

1R

$HO^{-}(H_2O_w)$

O	-1.24219400	0.09155400	-0.06787700
H	-1.56521700	-0.57603300	0.55085100
O	1.23693900	-0.09759200	-0.05373300
H	1.48506900	0.65863700	0.49001200
H	0.12219200	-0.03430500	-0.06798300

CH_3CH_2Br

C	-2.09358200	-0.40228700	-0.00000100
H	-2.01114300	-1.03744200	-0.89091400
H	-3.09120700	0.06466900	0.00021900
H	-2.01087100	-1.03770200	0.89070800
C	-1.03853300	0.68793700	-0.00000100
H	-1.08696000	1.31742800	-0.89364800
H	-1.08693500	1.31741700	0.89365900
Br	0.80228000	-0.06680800	0.00000000

anti-E2

1RC(aE)

C	0.50288800	1.45902800	0.54201400
H	-0.01877200	2.34725800	0.15781000
H	1.59566300	1.55530800	0.37274200
H	0.30323300	1.36259800	1.61809600
C	0.11112300	0.21131600	-0.20441900
H	0.26875400	0.29838100	-1.28158700
H	0.60924800	-0.68451900	0.17625700
Br	-1.88870700	-0.19300200	-0.05237900
O	3.29952600	0.75926800	-0.45824400
H	4.24830200	0.93216400	-0.47102500
O	2.86791900	-1.55971200	0.40419500
H	3.09649100	-0.56624000	0.04805200
H	2.97820500	-2.10837400	-0.38025300

1TS(aE)

C	0.61224300	0.65178700	0.59236600
H	0.35333600	1.70005300	0.79299600
H	1.79222500	0.67182500	0.11302500
H	0.67537700	0.07497100	1.52356100
C	-0.18995300	0.03401600	-0.43638000
H	-0.31934200	0.59936100	-1.36000600
H	-0.02076900	-1.02852800	-0.61456500

Br	-2.41537500	-0.15960300	0.01551600
O	3.11238800	0.70669200	-0.36753400
H	3.38319800	1.63334900	-0.38764400
O	5.19489500	-0.79257000	0.23990600
H	4.39032300	-0.20221500	0.05342100
H	5.29177000	-1.29050300	-0.57874100

1PC(aE)

C	3.43470200	-0.65087200	0.06813800
H	3.13343200	-1.22178000	0.94880700
H	1.39306700	1.23533700	1.07714200
H	4.48373200	-0.34964500	0.01082700
C	2.55251400	-0.33777500	-0.88741600
H	1.49982400	-0.63008700	-0.82168000
H	2.85481800	0.23934300	-1.76463400
Br	-1.26178700	-0.88034000	0.01953400
O	0.55877900	1.52022800	1.47254200
H	-0.05267400	0.78815300	1.21880600
O	-0.97982500	2.54713200	-0.89743800
H	-0.42080600	2.52193300	-0.10251600
H	-1.28378400	1.62163800	-0.93560000

1P1(E)

H_2O

O	0.00000000	0.00000000	0.11782600
H	0.00000000	0.76415700	-0.47130400
H	0.00000000	-0.76415700	-0.47130400

$CH_2=CH_2$

C	0.00000000	0.66763200	0.00000000
H	0.92871600	1.24080900	0.00000000
H	-0.92871600	1.24080900	0.00000000
C	0.00000000	-0.66763200	0.00000000
H	-0.92871600	-1.24080900	0.00000000
H	0.92871600	-1.24080900	0.00000000

syn-E2

1RC(sE)

C	-0.56845200	1.39071200	-0.31544500
H	-1.64875000	1.44117900	-0.09809200
H	-0.44768800	1.31160600	-1.40400700
H	-0.07326700	2.30861400	0.03404100
C	-0.04732300	0.15378600	0.37741800
H	-0.07539000	0.23722700	1.46788600

H	-0.57911500	-0.75289800	0.04820500
Br	1.93688100	-0.13090000	-0.00236600
O	-2.45125800	-1.44507200	-0.27437000
H	-2.84145800	-2.25811100	0.06789600
O	-3.88785500	0.53608800	0.27993900
H	-3.29717600	-0.34236600	0.06660400
H	-4.42042700	0.64113600	-0.51612100

1TS(sE)

C	0.40006100	1.78642500	0.14319800
H	0.93935300	2.29440500	-0.66954000
H	1.11817800	0.77544100	0.31465800
H	0.49325500	2.33898400	1.08850100
C	-0.99447900	1.52996700	-0.21697500
H	-1.77441600	1.92054300	0.43778700
H	-1.26006800	1.63367600	-1.26992700
Br	-1.65006200	-0.58895500	-0.02881800
O	2.03179700	-0.28547300	0.53499600
H	1.48742400	-1.07356700	0.41246100
O	4.45372000	-0.48986200	-0.42884900
H	3.48609000	-0.42067400	-0.10920600
H	4.94473800	-0.55105700	0.39740400

1PC(sE)

C	3.43558900	-0.64887500	-0.06829200
H	4.48440200	-0.34702800	-0.01024500
H	1.39293600	1.23803100	-1.07479700
H	3.13497800	-1.21875800	-0.94985000
C	2.55287600	-0.33771600	0.88741200
H	1.50039500	-0.63064100	0.82096400
H	2.85456400	0.23837400	1.76552000
Br	-1.26106700	-0.88125500	-0.01992500
O	0.55865200	1.52211300	-1.47078900
H	-0.05219500	0.78917000	-1.21812100
O	-0.98345300	2.54680200	0.89718200
H	-0.42348300	2.52196700	0.10290900
H	-1.28663600	1.62103900	0.93514800

inv-S_N2

1RC(iS)

C	0.56899500	-1.38979800	0.31488500
H	0.44845800	-1.31083000	1.40348400
H	1.64926500	-1.43978000	0.09728100
H	0.07412600	-2.30788300	-0.03456800

C	0.04713900	-0.15303400	-0.37770900
H	0.57856100	0.75381500	-0.04844000
H	0.07503100	-0.23627000	-1.46820000
Br	-1.93714600	0.13063000	0.00252900
O	2.45147000	1.44486000	0.27387000
H	2.84240100	2.25791200	-0.06753200
O	3.88833800	-0.53634000	-0.27945000
H	4.41939000	-0.64236400	0.51751100
H	3.29761500	0.34217000	-0.06648000

1TS(iS)

C	-0.60735100	1.24168300	0.16166800
H	-0.19885400	1.70606000	1.06813700
H	-1.70390600	1.27221900	0.19656300
H	-0.25686400	1.80933500	-0.70938200
C	-0.19786700	-0.19873700	0.06438500
H	-0.19480800	-0.81190700	0.95248100
H	-0.29156800	-0.71147300	-0.88164500
Br	2.14564600	-0.06959800	-0.04922200
O	-2.38907900	-0.90528100	0.20449700
H	-2.43346400	-1.85435500	0.03119400
O	-4.66171400	0.24073700	-0.29708300
H	-5.01900000	0.31521000	0.59430100
H	-3.76148400	-0.23046500	-0.14449300

1PC(iS)

C	3.51506600	0.42519900	0.20289500
H	3.94826100	0.75510500	-0.75299900
H	3.66145100	-0.66111000	0.28893500
H	4.06157800	0.91667300	1.02268100
C	2.03356600	0.76994900	0.26655000
H	1.89798500	1.86581100	0.21065600
H	1.60225700	0.43617800	1.22548000
Br	-1.79305200	0.34272000	0.04416000
O	1.37205900	0.13481000	-0.81937300
H	0.39973000	0.27407900	-0.69800600
O	0.31542300	-2.50187800	0.11818300
H	-0.49747100	-1.97833400	0.24714800
H	0.89139700	-1.83794100	-0.29665000

1P1(S)

CH_3CH_2OH

C	1.21761000	-0.24251800	0.02172500
H	2.08631100	0.42806900	-0.05724200

H	1.28009700	-0.96835200	-0.80378100
H	1.28465500	-0.79208500	0.97057000
C	-0.07840100	0.55697300	-0.04723700
H	-0.13034900	1.12497700	-0.99269100
H	-0.12723300	1.28192900	0.77618800
O	-1.24513300	-0.25713400	0.10842300
H	-1.26767400	-0.90419900	-0.60736000

ret-S_N2

1RC(rS)

C	-0.56845800	1.39079300	0.31568800
H	-0.44760700	1.31161000	1.40423500
H	-1.64877100	1.44132600	0.09841800
H	-0.07325400	2.30869100	-0.03377800
C	-0.04745300	0.15388500	-0.37730200
H	-0.57927300	-0.75280100	-0.04812300
H	-0.07559600	0.23741400	-1.46776300
Br	1.93677200	-0.13093100	0.00230400
O	-2.45129800	-1.44525500	0.27464400
H	-2.84114600	-2.25800100	-0.06871900
O	-3.88726300	0.53621400	-0.28015100
H	-3.29683500	-0.34237400	-0.06662100
H	-4.42058600	0.64096900	0.51544300

1TS(rS)

C	-0.64809000	2.15159900	0.28629700
H	-1.52578500	1.97106900	0.91028000
H	-0.76140300	3.09506100	-0.28881100
H	0.23437600	2.27701200	0.92695600
C	-0.44481300	1.05126500	-0.69158600
H	-1.30498900	0.50059400	-1.05051100
H	0.36368400	1.18705700	-1.40227700
Br	1.67978700	-0.41022600	-0.02894300
O	-1.49893400	-0.42015200	0.84106700
H	-0.80643400	-1.09158000	0.87509300
O	-3.66550500	-0.97275000	-0.44398600
H	-2.78113900	-0.82315500	0.06626300
H	-4.33791100	-0.83211500	0.23111200

1PC(rS)

C	-2.36150600	0.61723200	1.07175200
H	-2.77924800	-0.37758600	1.28392900
H	-3.01121700	1.37816800	1.53527200
H	-1.36414400	0.67683200	1.52751400

C	-2.26405000	0.85011600	-0.43577200
H	-3.26385400	0.79300200	-0.89649800
H	-1.86736100	1.86313500	-0.62816100
Br	1.59241300	0.36404100	0.04118900
O	-1.46407800	-0.12041100	-1.09792700
H	-0.51973600	0.05571200	-0.85967300
O	-0.36151800	-2.61551500	0.18969800
H	-0.97742000	-2.01973000	-0.26735400
H	0.40662700	-2.02765700	0.31331700

PT

1RC(PT)

C	-0.53785400	1.44864300	0.38182300
H	-0.40760800	1.33551100	1.46635300
H	-1.61728100	1.54620900	0.17867500
H	-0.01035300	2.35553600	0.05123500
C	-0.07413300	0.21147200	-0.35102500
H	-0.62670200	-0.68776500	-0.03107700
H	-0.11979800	0.32367000	-1.43833600
Br	1.90166400	-0.15102400	-0.01460000
O	-2.44078900	-1.51583600	0.16784900
H	-2.82433800	-2.06573700	0.86129900
O	-3.77733700	0.57610400	-0.14926000
H	-4.30844500	0.38000900	-0.92860700
H	-3.22678600	-0.34443500	0.01796500

1TS(PT)

C	-0.08463900	2.23967100	0.55441700
H	-0.05941500	2.60161500	-0.48521100
H	0.67009400	2.82512300	1.11409700
H	-1.07813800	2.49103600	0.97771400
C	0.34440000	0.78147300	0.65584800
H	1.87394100	0.69268000	-0.28875400
H	0.16769400	0.44999600	1.69581700
Br	-1.23188000	-0.34512200	-0.25526400
O	2.75216100	0.72432900	-0.82803300
H	2.71184500	-0.07629700	-1.36427000
O	1.60466500	-2.17440600	0.69327000
H	0.68186400	-2.22325900	0.40242400
H	1.73474300	-1.20788000	0.69893100

1PC(PT)

C	0.16328100	2.10122400	0.46647000
H	0.31836300	2.36450200	-0.59146000

H	0.97058000	2.59457300	1.04043300
H	-0.79599100	2.55339200	0.78845700
C	0.30680900	0.60178400	0.71045600
H	2.00469500	0.57076000	-0.37421700
H	0.01134200	0.40854000	1.75845500
Br	-1.38381700	-0.29535200	-0.22443800
O	2.86078900	0.56533300	-0.88332500
H	3.12651500	-0.35613300	-0.76347300
O	1.81915300	-1.89128000	0.64405500
H	1.15377100	-2.42647100	0.19581000
H	1.38423400	-0.98232300	0.65393900

1P1(PT)

CH_3CHBr^-

C	2.12917500	-0.37743400	0.02190100
H	2.11917400	-1.07515700	-0.83254200
H	3.14957100	0.05591100	0.06558300
H	1.99329800	-0.98749500	0.94530400
C	1.18894100	0.80679100	-0.19696400
H	1.15492600	1.36250700	0.76594800
Br	-0.80930500	-0.05519700	0.00303100

Dits

1TS(Dits)

C	0.43504000	1.84165900	0.03634900
H	1.13574500	1.38657200	-0.71546200
H	-0.13163200	2.61319800	-0.52949300
H	1.06920500	2.39109000	0.75550600
C	-0.41834200	0.90773100	0.82088100
H	-0.26361700	0.55764100	1.83485700
H	0.98549300	-0.44922400	0.85244000
Br	-1.58344900	-0.30138700	-0.22720100
O	1.67770000	-1.18593200	0.75119400
H	1.21528700	-1.81571300	0.18533200
O	3.90479400	-0.15165200	-0.56799300
H	3.49550500	0.62457100	-0.96910300
H	3.15459900	-0.55524800	-0.07104400

Complex

$Br^-(H_2O)$

Br	-0.72445100	0.00230800	-0.00000300
O	2.62915300	-0.08912000	-0.00003600
H	2.66905100	0.87407200	0.00006000
H	1.65352900	-0.24188200	0.00032400

$Br^{-}(CH_2=CH_2)$

Br	1.36030200	-0.02658800	-0.00002100
C	-2.51057100	0.53849400	0.00017500
H	-2.80282700	1.59270900	-0.00034600
H	-1.43485200	0.32472400	0.00082900
C	-3.43997300	-0.42322500	-0.00006100
H	-3.15903000	-1.47883900	0.00042800
H	-4.51059600	-0.19963400	-0.00087000

$CH_2=CH_2(H_2O)$

O	-2.42342900	-0.04989100	0.00034000
H	-2.96719500	-0.25975100	-0.76842900
H	-2.97620300	-0.26400700	0.76147000
C	2.01966300	-0.44757200	-0.00032300
H	3.09798500	-0.27733200	-0.00340800
H	1.68923200	-1.48802600	0.00178800
C	1.14582200	0.56303000	0.00085300
H	0.06765000	0.39388000	0.00393500
H	1.48305400	1.60162200	-0.00125800

$Br^{-}(H_2O)$ (H_2O)

Br	1.09047300	-0.04833000	0.00145800
O	-2.18347000	-1.44875600	0.01011800
H	-2.37041300	-0.49999000	-0.08706400
H	-1.20875200	-1.43783800	0.03669800
O	-1.80230600	1.54082000	-0.09004200
H	-1.80345400	1.73547800	0.85488400
H	-0.89773300	1.15738100	-0.21614300

$Br^{-}(CH_2=CH_2)$ (H_2O)

Br	1.45723100	-0.31731700	-0.00762500
C	-2.46582700	-1.25554800	0.06721400
H	-2.90635500	-2.25197700	0.16784300
H	-1.37404400	-1.18264800	0.07965900
C	-3.23408800	-0.16941800	-0.06903100
H	-2.78397000	0.82088600	-0.16965300
H	-4.32602400	-0.23572700	-0.08527100
O	-0.61287900	2.31364600	-0.05020100
H	-0.01062400	1.54739000	-0.21099100
H	-0.49955600	2.44878300	0.89779800

$CH_2=CH_2(H_2O)$ (H_2O)

C	-2.07623100	0.02042400	0.48012900
H	-1.69041000	0.02657600	1.50130300

H	-2.97974900	-0.56524900	0.30135700
C	-1.48044700	0.71516900	-0.49665400
H	-1.87986900	0.71803000	-1.51275100
H	-0.57930500	1.30446700	-0.31659100
O	1.96148800	1.08800400	0.07776600
H	1.78097000	0.12999900	0.08405600
H	2.75303200	1.20343500	0.61469300
O	0.89889600	-1.57392100	-0.03085300
H	1.05718900	-2.11124700	-0.81622800
H	-0.00486400	-1.23222700	-0.13200100

$Br^{-}(CH_3CH_2OH)$

C	-3.57925600	0.17847800	0.01029700
H	-3.87715600	-0.37498600	0.91340300
H	-3.89867100	-0.40520200	-0.86608900
H	-4.10965000	1.14373500	0.00019700
C	-2.07063000	0.39086100	-0.01200300
H	-1.78350100	0.96823400	-0.91078600
H	-1.75964300	0.99067400	0.86355800
O	-1.43740500	-0.87367000	-0.00411300
H	-0.46175700	-0.71795300	-0.00472500
Br	1.75111200	0.05625200	0.00135900

$CH_3CH_2OH(H_2O)$

O	-2.79320100	0.17635000	-0.00046700
H	-3.22411500	-0.22334300	-0.76645900
H	-3.22538000	-0.22093300	0.76602900
C	2.35413600	-0.06789100	-0.00060200
H	2.52227000	-0.68919000	-0.89114900
H	2.52333500	-0.68963400	0.88943400
H	3.08935000	0.74965500	-0.00083500
C	0.94034700	0.48917200	0.00038400
H	0.78450300	1.12358000	0.89169100
H	0.78338300	1.12390900	-0.89048700
O	0.02601400	-0.60597000	0.00075900
H	-0.88274100	-0.26476700	0.00074000

$CH3CHBr^{-}(H_2O)$

C	0.72207900	0.62298600	0.51065400
H	0.47061400	0.81702900	1.57080000
C	0.71732500	1.95451900	-0.22855400
H	1.60614800	2.51227300	0.12625500
H	0.84117200	1.81696400	-1.31498500
H	-0.16632900	2.60840500	-0.07220300

Br	-1.09743600	-0.34816500	-0.03297000
O	2.82738000	-1.08903900	-0.07471900
H	2.02368100	-0.44649200	0.15788200
H	2.37950800	-1.87512200	-0.40866300

(H_2O) (H_2O)

O	1.38749200	0.00117800	0.11112000
H	1.74443300	-0.76986500	-0.34742100
H	1.74307100	0.76295100	-0.36366900
O	-1.51406700	-0.00157200	-0.12179700
H	-1.91944300	0.00984100	0.75244200
H	-0.55546300	0.00023000	0.04406800

$HO^-(H_2O_w)_2 + CH_3CH_2Br$ (B3LYP):

2R

$HO^-(H_2O_w)_2$

O	-0.00000600	0.65359000	-0.01506900
H	0.00010400	1.44273800	0.53931000
O	-2.39113400	-0.29744300	-0.07849800
H	-2.29559400	-1.06515200	0.49517600
H	-1.43384800	0.10875600	-0.07640100
O	2.39097400	-0.29769500	-0.07897600
H	2.29701200	-1.06241300	0.49896100
H	1.43365500	0.10844900	-0.07669900

anti-E2

2RC(aE)

C	0.06495700	0.00004300	1.66169300
H	-0.34256600	0.89536000	2.15069100
H	1.16528100	0.00003300	1.75044000
H	-0.34260100	-0.89517400	2.15082100
C	-0.21344400	-0.00005200	0.17828800
H	0.16438900	0.89830900	-0.31589400
H	0.16430400	-0.89851100	-0.31577500
Br	-2.20860900	-0.00001100	-0.22008400
O	3.05682400	0.00003600	0.49127300
H	3.97216300	0.00000300	0.79182000
O	2.39731600	2.22773500	-0.62771600
H	2.60363200	2.06994200	-1.55522400
H	2.69568200	1.35348600	-0.16285300

O	2.39743600	-2.22773100	-0.62767800
H	2.69570300	-1.35344400	-0.16281500
H	2.60365300	-2.06988000	-1.55519900

2TS(aE)

C	0.03809400	-0.13999200	-1.17386200
H	-0.34463200	-0.47733300	-2.14524200
H	1.32385000	-0.49787900	-1.18222100
H	0.05881900	0.95345100	-1.09616300
C	-0.51082300	-0.81733300	-0.04302300
H	-0.61563200	-1.90040200	-0.10437600
H	-0.22536400	-0.46188000	0.94674000
Br	-2.80989700	-0.48310200	0.33995200
O	2.61007600	-0.78772000	-1.27083500
H	2.83736300	-0.70020100	-2.20485100
O	3.69646700	-3.02234200	-0.20464800
H	3.05814600	-3.28703800	0.46666700
H	3.26574900	-2.23582600	-0.64584900
O	4.71570300	0.07425200	0.30505900
H	3.92867900	-0.07357600	-0.27499100
H	4.95502100	-0.83353700	0.53269600

2PC(aE)

C	-3.28767100	1.91133700	-0.23457000
H	-3.12921900	1.55871700	-1.25562400
H	-0.38749000	1.94312200	-0.31316900
H	-3.82395300	2.85590400	-0.11585300
C	-2.84243000	1.21133400	0.81469100
H	-2.29735500	0.27099700	0.69010600
H	-3.00065800	1.56729600	1.83569400
Br	-0.26251900	-1.65076100	-0.23336400
O	0.47039100	1.55006700	-0.51586600
H	0.27157500	0.58229400	-0.57623400
O	2.23599800	0.01573800	1.48257700
H	1.71623300	-0.72260300	1.10513200
H	1.66717500	0.76976000	1.26471600
O	3.42647500	1.83144500	-0.61222000
H	2.48103300	1.94060600	-0.80723100
H	3.40850400	1.09650100	0.02353800

syn-E2

2RC(sE)

C	-0.19271100	-1.24131200	0.63096200

H	0.17651100	-2.19599700	0.23234000
H	-1.28546600	-1.20946700	0.51018300
H	0.05723600	-1.18354200	1.69902700
C	0.36490300	-0.06407200	-0.13270900
H	0.12601300	-0.10102400	-1.19604300
H	0.06334700	0.90062600	0.28094300
Br	2.40051800	-0.04080000	-0.06806900
O	-2.63020800	0.39672000	-0.89545100
H	-3.05218600	0.71482900	-1.70161100
O	-1.86551000	2.38914700	0.53549600
H	-2.49909700	2.40368200	1.26060200
H	-2.19534700	1.60735000	-0.06344100
O	-4.05403900	-1.51720700	0.09829000
H	-3.49781800	-0.75870100	-0.32858400
H	-4.54641900	-1.06674700	0.79280700

2TS(sE)

C	-0.13555700	-1.91077300	-0.25221200
H	0.63785700	-0.91319500	-0.06626900
H	0.22137600	-2.27853900	-1.22321100
H	0.15447200	-2.56876000	0.57732000
C	-1.54510100	-1.58342800	-0.25291900
H	-2.08328300	-1.60451600	-1.19985400
H	-2.15643300	-1.90835800	0.58813800
Br	-2.03788700	0.63781800	0.10834700
O	1.52683700	0.11367600	0.12888900
H	0.92352200	0.85547200	0.26667300
O	3.47397500	0.53762300	-1.65302400
H	3.49459700	1.49564100	-1.75026900
H	2.69526900	0.38013900	-1.03530800
O	3.78557200	-0.01453000	1.64676400
H	2.88195900	0.02415000	1.23331100
H	4.34960700	0.06540800	0.86709500

2PC(sE)

C	-2.79197200	2.24978700	0.65584200
H	-0.27947100	1.85861200	-0.73621400
H	-2.09949800	2.09154000	1.48489400
H	-3.29813200	3.21720100	0.61312300
C	-3.00463200	1.29698700	-0.25852700
H	-2.48989100	0.33257500	-0.21612500
H	-3.69808300	1.45868300	-1.08762900
Br	-0.34228600	-1.68656700	-0.12509500
O	0.57006100	1.40434700	-0.79534100

H	0.32464100	0.44656000	-0.73868300
O	2.03768800	0.18813600	1.57490200
H	1.53940800	-0.60087600	1.28235300
H	1.53133600	0.89287900	1.14147300
O	3.53987800	1.58231700	-0.64192400
H	2.62540000	1.68395500	-0.95319400
H	3.42290500	0.96967700	0.10333800

inv-*S_N*2

2RC(iS)

C	0.13973300	-1.28393100	1.15087500
H	0.89103800	-2.00078600	1.51178600
H	-0.82676000	-1.79609000	1.01589800
H	0.00569800	-0.49670700	1.90407300
C	0.50755700	-0.69481900	-0.19179800
H	0.70276700	-1.46172300	-0.94702300
H	-0.25346000	0.01255800	-0.55227100
Br	2.25988900	0.32478100	-0.10298500
O	-2.30808200	0.36782300	-0.87735000
H	-2.55690400	0.55857700	-1.78922700
O	-3.35370500	2.23336400	0.61541200
H	-2.56989300	2.72726800	0.87882000
H	-2.97077300	1.49076500	0.01914000
O	-2.95699100	-2.04346200	-0.23429000
H	-3.74148100	-1.90826400	0.30827500
H	-2.70988500	-1.08222600	-0.52964600

2TS(iS)

C	-0.20606800	-0.31541700	1.29704300
H	0.28054100	-1.23824000	1.63321800
H	-1.29168600	-0.42614700	1.41352000
H	0.14522200	0.51194600	1.92540200
C	0.09413800	-0.05371100	-0.14947800
H	0.11746300	-0.87153400	-0.85345100
H	0.07644400	0.94988100	-0.54382100
Br	2.52392500	0.01210000	-0.10674800
O	-2.03829700	0.03005400	-0.74919600
H	-2.02357300	0.09179600	-1.71281000
O	-3.36139500	2.17348400	0.12671600
H	-2.68426100	2.67532600	0.59274900
H	-2.86864000	1.36307100	-0.21651000
O	-3.60661700	-2.01088500	-0.04461300
H	-4.37622300	-1.53487800	0.28609800
H	-2.99061300	-1.27119300	-0.33687700

2PC(iS)

C	-3.58599800	-0.84139700	-0.24205200
H	-4.11241500	-1.34320300	-1.06852300
H	-3.83228600	0.22938600	-0.27056200
H	-3.95306800	-1.25506700	0.70870000
C	-2.08318700	-1.04487800	-0.36879200
H	-1.72251700	-0.63572400	-1.32800300
H	-1.83910000	-2.12187700	-0.35879700
Br	1.76760200	-0.80047700	0.01918700
O	-1.44358000	-0.38893300	0.72101900
H	-0.47035200	-0.51893500	0.62012600
O	1.35124800	2.46909500	-0.41819600
H	1.47850900	1.48983500	-0.44154400
H	1.72662400	2.70126200	0.43962500
O	-1.52655700	2.49938300	0.23131700
H	-0.60286400	2.59359800	-0.06627500
H	-1.57235500	1.55872000	0.48564800

ret-S$_N$2

2RC(rS)

C	0.14093300	-1.28557200	1.15035100
H	0.00708300	-0.49924000	1.90451100
H	0.89267300	-2.00258500	1.51003500
H	-0.82546300	-1.79790600	1.01532800
C	0.50779700	-0.69486200	-0.19188300
H	0.70296300	-1.46089900	-0.94799500
H	-0.25371300	0.01253800	-0.55127900
Br	2.25970400	0.32545300	-0.10293500
O	-2.30824600	0.36777200	-0.87640900
H	-2.55613600	0.55858800	-1.78852800
O	-3.35538900	2.23340500	0.61521400
H	-2.57177700	2.72690200	0.87997800
H	-2.97185100	1.49074700	0.01942400
O	-2.95576000	-2.04415900	-0.23462900
H	-2.70922200	-1.08258800	-0.52944200
H	-3.74142900	-1.90995600	0.30647000

2TS(rS)

C	-0.94791400	0.03340500	-1.98664300
H	-0.36634700	-0.89809500	-1.94291600
H	-0.85395200	0.42198200	-3.01968400
H	-1.98689100	-0.21034200	-1.74847600
C	-0.40188300	1.04599800	-1.05486600

H	0.50695800	1.55186800	-1.35918200
H	-1.07257100	1.60827200	-0.41692100
Br	1.83982900	0.30606500	0.31865000
O	-1.37195400	-0.24319900	0.68157700
H	-0.71182300	-0.00749100	1.34571900
O	-0.44930500	-2.66917700	-0.03368000
H	0.48896900	-2.45648000	0.05725400
H	-0.87430600	-1.80983600	0.25835200
O	-3.71726100	0.90691600	0.85082400
H	-2.80265600	0.46404000	0.84018500
H	-4.31443500	0.19107600	1.09221000

2PC(rS)

C	-3.61835500	-0.00011100	-0.45350300
H	-3.86320400	-0.89072600	0.14291900
H	-4.24843600	-0.00021800	-1.35595400
H	-3.86372100	0.89025800	0.14307200
C	-2.14475200	0.00035600	-0.83220900
H	-1.90573600	-0.88919500	-1.44030300
H	-1.90630900	0.89010300	-1.44025000
Br	1.79525500	-0.00030200	-0.39677600
O	-1.36844700	0.00055900	0.36222500
H	-0.41023500	0.00038600	0.10372900
O	-0.06597500	-2.62636100	1.17052800
H	0.70663200	-2.16948100	0.79151700
H	-0.73942500	-1.92975800	1.12108700
O	-0.06480000	2.62677100	1.17018900
H	-0.73866000	1.93057400	1.12079200
H	0.70759800	2.16942700	0.79128200

$HO^{-}(H_2O_w)_3 + CH_3CH_2Br$ (B3LYP):

3R

$HO^{-}(H_2O_w)_3$

O	0.00524700	-0.00087200	0.65450800
H	0.00551500	-0.00088700	1.61855600
O	2.48082500	-0.20021000	-0.21823200
H	2.56066000	0.63045600	-0.69959300
H	1.53127300	-0.15563400	0.14578500
O	-1.41728700	-2.03764100	-0.21664900
H	-0.73925800	-2.53031900	-0.69171100

H	-0.89901500	-1.24036600	0.14647100
O	-1.06905500	2.23984500	-0.21580100
H	-1.82746200	1.89129000	-0.69695300
H	-0.62955300	1.39648200	0.14683900

anti-E2

3RC(aE)

C	0.67016900	0.03514400	1.70247300
H	1.13360400	-0.85259200	2.15220600
H	-0.40155200	0.04151200	1.95180300
H	1.13544100	0.93940600	2.11586200
C	0.75643200	0.00435800	0.19420000
H	0.31794100	-0.90469600	-0.22396800
H	0.32041500	0.89686200	-0.26046700
Br	2.68064100	-0.01053900	-0.44031800
O	-2.81496200	0.01707100	0.72460700
H	-3.32625900	0.03056100	1.54150400
O	-1.69257800	-2.31443100	0.30099000
H	-2.12720000	-1.41847800	0.50640200
H	-2.31443900	-2.72559800	-0.30891600
O	-1.68576800	2.32979800	0.23798200
H	-2.12554600	1.44052300	0.46474900
H	-2.32444100	2.75044300	-0.34740200
O	-4.43421700	-0.03997900	-1.37829200
H	-3.85614800	-0.01839500	-0.54702900
H	-3.79367100	0.01261900	-2.09594200

3TS(aE)

C	-0.32206200	-0.00801800	-1.19230100
H	-0.46972400	-0.92684700	-1.77067300
H	1.04193000	-0.00383100	-0.94287900
H	-0.47249200	0.90085600	-1.78553000
C	-0.88138700	0.00179900	0.10779300
H	-0.82919900	-0.90855000	0.70266200
H	-0.83145400	0.92175000	0.68785700
Br	-3.30906000	-0.00008700	0.21887000
O	2.30031700	-0.00103100	-0.82105500
H	2.65898900	-0.00175800	-1.71820600
O	3.17301600	-2.68531900	-0.15391100
H	2.72869700	-1.86340700	-0.44807000
H	3.83050400	-2.34132600	0.46535700
O	3.16348300	2.68767200	-0.16010800
H	2.72215100	1.86353200	-0.45249700
H	3.82242100	2.34736000	0.45966100

O	4.09463000	0.00435000	1.27154200
H	3.46204100	0.00231100	0.50962100
H	3.52237000	0.00491100	2.04755800

3PC(aE)

C	-3.48407600	1.54915700	0.41535000
H	-4.13329300	2.42066300	0.30236800
H	-0.89394200	2.16732500	-0.04056000
H	-3.16769600	1.29925500	1.42999000
C	-3.08901100	0.81674700	-0.63233300
H	-3.40628500	1.07207200	-1.64650200
H	-2.42949000	-0.04894200	-0.51657000
Br	-0.24651000	-1.81612000	0.05045200
O	0.03471100	2.43516900	-0.00070400
H	0.41779700	1.87032300	0.71491400
O	3.57396100	0.90549800	-0.04990400
H	3.71072600	1.85752200	-0.11639500
H	2.87105400	0.71852000	-0.72504500
O	1.25921800	0.67533400	1.76866400
H	0.81869500	-0.13950300	1.43636100
H	2.13343600	0.65477400	1.33586800
O	1.41765500	0.54722400	-1.71284000
H	0.88247100	1.25698600	-1.30407000
H	0.97854900	-0.26601500	-1.37599500

syn-E2

3RC(sE)

C	-0.49769500	-1.09412400	-0.14352500
H	-0.67389900	-1.59389400	0.81793800
H	0.58466200	-0.90204100	-0.24344200
H	-0.81878600	-1.76359400	-0.95293700
C	-1.20695300	0.23802600	-0.20719700
H	-0.89997800	0.92112200	0.58665500
H	-1.09649200	0.73350300	-1.17489100
Br	-3.21061500	0.03884100	0.00531800
O	2.77271900	0.01523300	-0.55674000
H	2.62251500	-0.00802000	-1.50887100
O	3.53417400	-2.32042900	0.41772000
H	3.24110000	-1.44245500	0.00592700
H	3.12723100	-2.30354200	1.29059500
O	1.35487600	1.84041000	0.66802500
H	1.89085300	1.13009600	0.17288100
H	2.02795300	2.47314400	0.94006200
O	5.17328900	1.13896200	-0.24611600

H	4.25361300	0.75027400	-0.39272000
H	5.66018800	0.38915800	0.11390500

3TS(sE)

C	0.37584400	-1.65041200	0.96358500
H	0.05620400	-1.60886400	2.01226100
H	-0.02009100	-2.51730600	0.42066900
H	-0.35288200	-0.70750500	0.43999100
C	1.78064200	-1.43100800	0.77369600
H	2.39751900	-1.14653600	1.62381100
H	2.31193900	-2.01191600	0.02231000
Br	2.34152300	0.59051800	-0.33682000
O	-1.14018000	0.23422000	-0.09163100
H	-0.44732300	0.82816500	-0.41436200
O	-2.68141800	0.80275600	2.06991300
H	-2.03023100	0.66545500	1.32659900
H	-2.88741700	-0.09409500	2.35628700
O	-2.65686500	-1.64579000	-1.59386400
H	-1.97458000	-1.08279900	-1.16792700
H	-3.43121200	-1.06694000	-1.55067200
O	-3.74283900	1.15989100	-0.77710400
H	-2.78927700	0.92767000	-0.72833900
H	-3.95443600	1.22643800	0.16585700

3PC(sE)

C	-3.53205200	1.98648000	0.45944300
H	-3.06084200	1.79833100	1.42612400
H	-4.08316500	2.92378200	0.35306200
H	-0.77235800	1.88598300	-0.27372600
C	-3.43508400	1.10387200	-0.54096400
H	-2.87518200	0.17046500	-0.43404300
H	-3.90738400	1.29514000	-1.50754300
Br	-0.62913900	-1.69088800	0.05800700
O	0.09768900	1.47505200	-0.19658300
H	-0.11074800	0.50624600	-0.12663300
O	2.51768400	1.80816900	1.37968000
H	1.57168500	1.79010300	1.14660200
H	2.92324900	1.86854700	0.49633600
O	2.77492400	1.27436500	-1.51739400
H	1.82711500	1.46233600	-1.41017200
H	2.84820300	0.37038200	-1.14581200
O	2.73108300	-1.02121400	0.19664800
H	1.81812100	-1.37626200	0.14161800
H	2.67294000	-0.34707900	0.89427700

***inv*-S$_N$2**

3RC(iS)

C	1.03763400	2.10690200	0.41913600
H	1.24169800	1.98045400	1.49058400
H	0.11246100	2.69146400	0.30396600
H	1.87360300	2.65445200	-0.03793500
C	0.79727600	0.77336400	-0.25347000
H	-0.02913500	0.21452200	0.20189900
H	0.63554400	0.86917200	-1.32995200
Br	2.41748800	-0.42769500	-0.11120300
O	-2.14731100	-0.16160500	0.78655800
H	-2.29788500	-0.13597000	1.73854000
O	-1.37907800	-2.62976300	0.13620000
H	-0.42870300	-2.53898300	0.00408900
H	-1.67684500	-1.70238700	0.39941400
O	-4.49296800	-0.48694100	-0.44898500
H	-4.34269100	-1.32258100	-0.90520100
H	-3.63236700	-0.35678300	0.06012900
O	-2.21375900	2.25212600	-0.27830400
H	-3.04611100	2.19876100	-0.76162500
H	-2.16619500	1.34507700	0.17045700

3TS(iS)

C	0.50635200	1.75936000	0.74044200
H	1.10535400	1.84442200	1.65519700
H	-0.47806400	2.21616700	0.90451700
H	1.00933700	2.31638000	-0.05760500
C	0.33161600	0.32089300	0.34623600
H	0.41371900	-0.47569000	1.06819800
H	0.06062000	0.06307200	-0.66631200
Br	2.71329900	-0.21392900	-0.23906900
O	-1.77756400	0.10107900	0.67602400
H	-1.88586200	0.24133000	1.62571200
O	-1.68953800	-2.58778500	0.22539300
H	-0.84373600	-2.73747800	-0.21188600
H	-1.69862400	-1.61150000	0.42200600
O	-4.22908000	-0.79115000	-0.38180500
H	-3.94082900	-1.69538500	-0.56335600
H	-3.42075800	-0.40603900	0.02820200
O	-2.83921800	2.26399000	-0.66001500
H	-3.60918800	1.84896200	-1.06683800
H	-2.42203700	1.51268900	-0.16727600

3PC(iS)

C	-0.94803700	3.15595200	0.12155800
H	0.07192000	3.07491200	0.52125400
H	-1.65416800	3.17834500	0.96432400
H	-1.03289400	4.10492400	-0.43207200
C	-1.24749500	1.97583700	-0.79948100
H	-0.54686100	1.97702300	-1.65115400
H	-2.26928600	2.04934900	-1.20104300
Br	2.02676600	0.13945800	0.16146900
O	-1.17569600	0.72519200	-0.11784400
H	-0.22034100	0.53484000	0.06410800
O	0.66535100	-2.87758000	-0.13984500
H	1.15810900	-2.02219100	-0.17084000
H	0.50654100	-2.99054700	0.80529400
O	-1.89006500	-1.93698500	-1.09035900
H	-1.04955500	-2.37483200	-0.84700600
H	-1.67175200	-0.99239800	-0.98650800
O	-3.27346000	-0.96502900	1.29889200
H	-3.00266300	-1.55334500	0.56991700
H	-2.66171500	-0.22262500	1.17311200

ret-S_N2

3RC(rS)

C	1.03771100	2.10699100	-0.41870800
H	0.11256100	2.69156200	-0.30338600
H	1.24169500	1.98071000	-1.49019100
H	1.87373600	2.65443300	0.03839100
C	0.79735300	0.77334600	0.25368800
H	0.63575800	0.86897200	1.33020800
H	-0.02914500	0.21463000	-0.20168700
Br	2.41748000	-0.42778300	0.11103600
O	-2.14718400	-0.16141800	-0.78637700
H	-2.29762100	-0.13581700	-1.73838100
O	-2.21384900	2.25243500	0.27815700
H	-2.16623800	1.34530400	-0.17044200
H	-3.04617700	2.19914100	0.76152200
O	-1.37917000	-2.62961200	-0.13588100
H	-1.67682100	-1.70221200	-0.39913400
H	-0.42878600	-2.53893900	-0.00376600
O	-4.49304200	-0.48731700	0.44866400
H	-3.63235800	-0.35689400	-0.06024200
H	-4.34284500	-1.32320400	0.90445000

3TS(rS)

C	-0.47866900	-2.43352300	0.53843400
H	-0.32164700	-3.18105600	1.34630700
H	0.32048300	-2.60139500	-0.19063600
H	-1.46486400	-2.59287000	0.09256800
C	-0.36370200	-1.11886000	1.20058700
H	-1.23250600	-0.70150500	1.69610300
H	0.60310300	-0.80708300	1.56961700
Br	-2.05161600	0.39203200	-0.43617600
O	1.22939600	0.04193700	-0.21873300
H	0.55406000	0.19774900	-0.89277000
O	2.71777700	-2.14483100	-0.43844500
H	2.15572500	-1.31181400	-0.40869100
H	3.60979100	-1.80861200	-0.57675200
O	0.82875000	2.16797600	1.43506400
H	1.00685000	1.39455600	0.83281200
H	-0.08544400	2.38605300	1.21339400
O	3.45398200	1.59693100	-0.67379000
H	2.66764200	0.99760800	-0.59248700
H	3.20834000	2.32545500	-0.09020800

3PC(rS)

C	-3.07024100	1.25693300	-0.50989700
H	-3.86760700	1.96728200	-0.23866000
H	-3.49968600	0.47437000	-1.15136300
H	-2.30466100	1.79615500	-1.08435300
C	-2.45570400	0.64379700	0.74618300
H	-2.04409100	1.43883500	1.39104300
H	-3.22105700	0.10423800	1.32412000
Br	1.31653400	1.28793000	-0.30700300
O	-1.43975800	-0.30871600	0.44478700
H	-0.65473400	0.18182800	0.09295800
O	-1.16293300	-2.89467500	-1.04721900
H	-1.46215400	-2.07672400	-0.61753900
H	-0.19145800	-2.80723700	-0.99871900
O	0.93303800	-1.13885700	2.08892800
H	-0.00350600	-1.09915600	1.83189500
H	1.28342200	-0.33407500	1.65669300
O	1.64798800	-2.24280700	-0.54391900
H	1.70000200	-1.31425400	-0.83585800
H	1.51582800	-2.13274900	0.41653000

HO^-(PCM, H_2O) + CH_3CH_2Br (B3LYP):

R

HO^-

O	0.00000000	0.00000000	0.10745700
H	0.00000000	0.00000000	-0.85965500

CH_3CH_2Br

C	-2.09823100	-0.40538100	0.00000000
H	-2.01949900	-1.03780600	-0.89303500
H	-3.09267700	0.06719700	0.00000600
H	-2.01949200	-1.03781400	0.89302800
C	-1.05118600	0.68902700	0.00000000
H	-1.09107700	1.31482800	-0.89543900
H	-1.09107700	1.31482800	0.89543900
Br	0.80600900	-0.06637500	0.00000000

anti-E2

RC(aE)

C	0.29516400	-0.39805500	-0.00109800
C	1.07938200	0.89710200	-0.02634300
H	0.46527400	-1.01477700	-0.88737700
H	0.47410400	-0.98502800	0.90351100
H	0.86901700	1.51231900	0.85731600
H	2.15264100	0.64903200	-0.02778400
H	0.86148200	1.48206100	-0.92866500
O	5.15439700	-0.33282500	-0.03408400
H	4.63510700	0.35508000	0.40410200
Br	-1.68400200	-0.06658200	0.00332000

TS(aE)

C	0.63644900	-0.48047800	0.00282200
C	1.49938000	0.67440000	-0.04060400
H	0.60336700	-1.11007000	-0.88684500
H	0.61810200	-1.05153500	0.93154900
H	1.43351800	1.31980600	0.84481600
H	2.67340200	0.20435500	-0.02630900
H	1.42447500	1.25851200	-0.96659400
O	4.02180400	-0.25689100	-0.05829800
H	4.50319800	0.25473800	0.60645900
Br	-1.60701300	0.00045100	0.00542900

PC(aE)

C	3.54640500	0.23374200	-0.22365300
C	4.22261000	1.34080200	0.10563700
H	3.40546200	-0.06238800	-1.26458900
H	3.10094300	-0.41175100	0.53528900
H	4.35561700	1.64179200	1.14622300
H	5.93905000	-0.48159400	0.01319700
H	4.65996200	1.99135600	-0.65374000
O	6.78007300	-0.96422100	-0.02939500
H	6.83504400	-1.42530000	0.81756800
Br	-3.69002100	-0.08530300	0.00998000

P1(E)

H_2O

O	0.00000000	0.00000000	0.11868500
H	0.00000000	0.76259700	-0.47474000
H	0.00000000	-0.76259700	-0.47474000

$CH_2=CH_2$

C	0.00000000	0.00000000	0.66833900
H	0.00000000	0.92921000	1.24125500
H	0.00000000	-0.92921000	1.24125500
C	0.00000000	0.00000000	-0.66833900
H	0.00000000	-0.92921000	-1.24125500
H	0.00000000	0.92921000	-1.24125500

syn-E2

RC(sE)

C	-0.60192900	1.77802700	-0.36578500
H	-0.86609100	2.78919700	-0.01908900
H	-1.49575800	1.14606100	-0.28888000
H	-0.30044700	1.84448800	-1.41851200
C	0.52434100	1.25742800	0.50266000
H	1.43424100	1.85794900	0.42342000
H	0.23689100	1.15024600	1.55181500
Br	1.09276500	-0.58499900	-0.04993000
O	-4.19417800	-0.71346800	0.08479000
H	-3.23667200	-0.81795700	-0.00076800

TS(sE)

C	-1.81863400	1.06505400	-0.00210200
H	-2.35624400	1.37160500	0.90547900
H	-1.94620100	-0.17157400	-0.00098900
H	-2.35064600	1.37131600	-0.91300900
C	-0.43970300	1.51655500	0.00237300

H	-0.03117400	1.96004800	-0.90420600
H	-0.03576700	1.95396700	0.91394800
Br	1.10873200	-0.20872000	-0.00031600
O	-2.16199200	-1.59772200	0.00058700
H	-1.23963200	-1.88802500	0.00349500

PC(sE)

C	3.66976100	-0.51553200	0.66481600
H	3.11927500	-1.29223800	1.19849700
H	1.66105200	0.95995200	0.00994100
H	4.20278500	0.21583500	1.27485400
C	3.69642300	-0.46709700	-0.67207300
H	4.25232100	0.30568200	-1.20589600
H	3.16876000	-1.20233700	-1.28206200
Br	-1.94913800	-0.19937500	-0.00306300
O	0.93693400	1.60341300	0.01792600
H	0.12306600	1.05970600	0.01201900

inv-S_N2

RC(iS)

C	-0.40317400	1.88181800	0.16793600
H	-0.37213600	1.84118200	1.26383800
H	-1.35997300	2.33642600	-0.13210200
H	0.40978300	2.52636900	-0.18872900
C	-0.32386700	0.49780800	-0.44190100
H	-1.10926300	-0.17083400	-0.08003900
H	-0.32107100	0.51511000	-1.53483500
Br	1.38835500	-0.42686400	0.04673100
O	-4.52964300	-0.77361000	0.11026900
H	-5.24038400	-0.19688500	-0.20208600

TS(iS)

C	1.24928100	1.39237900	0.00006100
H	0.84470400	1.88556200	-0.89210200
H	2.34180800	1.49957600	-0.00038300
H	0.84544400	1.88563800	0.89252200
C	0.94323700	-0.07616900	0.00017900
H	1.04160500	-0.63698200	-0.91803500
H	1.04118000	-0.63681200	0.91861100
Br	-1.38372300	-0.14318400	-0.00003000
O	3.23853400	-0.65722400	-0.00029600
H	3.25219900	-1.62500100	0.00136100

PC(iS)

C	-3.70146600	1.15669200	-0.13020800
H	-3.27168700	2.08945700	0.26060700
H	-4.73661400	1.07590700	0.23019000
H	-3.71667300	1.21661400	-1.22752900
C	-2.87476000	-0.02853400	0.33323900
H	-2.85552500	-0.07680800	1.43430200
H	-1.83564700	0.06428600	-0.02212500
Br	2.47320300	0.01906100	-0.01116500
O	-3.46879700	-1.22786900	-0.19850900
H	-2.93824900	-1.98260400	0.08521900

P1(S)
CH_3CH_2OH

C	1.21708200	-0.24516500	0.02166500
H	2.08419200	0.42514200	-0.07122500
H	1.26851800	-0.97859900	-0.79684100
H	1.29344400	-0.78216400	0.97742900
C	-0.07411000	0.55913900	-0.04725700
H	-0.12964300	1.11681600	-0.99666100
H	-0.12023800	1.28787900	0.77266300
O	-1.24789900	-0.25713500	0.11031200
H	-1.27091400	-0.89583400	-0.61431400

ret-S_N2
RC(rS)

C	0.49823700	2.01003300	-0.23626300
H	0.60790900	1.85104800	-1.31673100
H	1.36626700	2.59251200	0.11125100
H	-0.40623200	2.60445200	-0.05410000
C	0.48063900	0.69704400	0.51891100
H	1.36013500	0.07041400	0.31835400
H	0.32895300	0.83417800	1.59330200
Br	-1.08694700	-0.43924500	-0.04390300
O	3.13236000	-0.91619800	-0.05817900
H	3.85398800	-1.49192100	-0.34591200

TS(rS)

C	-1.95171700	-1.31425900	-0.39835600
H	-2.30232400	-0.75648200	-1.27327400
H	-2.84361600	-1.83983500	0.01188900
H	-1.21151000	-2.07113000	-0.67336100
C	-1.53405200	-0.43654300	0.69795900
H	-2.07303600	0.48932800	0.88120800
H	-0.85978600	-0.81373200	1.46063300

Br	1.22837400	-0.06961200	-0.02064300
O	-1.52681800	2.00596900	-0.15287700
H	-0.57364900	1.88531600	-0.25920200

PC(rS)

C	2.75311300	1.00622600	-0.16528700
H	3.15826500	0.90686100	-1.18237400
H	3.49811700	1.52649900	0.45538100
H	1.84978200	1.63134900	-0.21003200
C	2.43951400	-0.36401500	0.42661200
H	3.35250000	-0.97406600	0.47478900
H	2.05996800	-0.25494100	1.45663600
Br	-1.65060000	0.08374400	0.01758000
O	1.50937000	-1.11324800	-0.36407700
H	0.62164500	-0.71403700	-0.26502500

PT

P1(PT)

CH_3CHBr^-

C	2.14259900	-0.37971000	0.02204700
H	2.13522700	-1.08819800	-0.82160100
H	3.16122400	0.05331100	0.05949300
H	2.00946200	-0.96488700	0.95422200
C	1.20987900	0.80363200	-0.19806800
H	1.16684500	1.35982700	0.76066300
Br	-0.81678900	-0.05438800	0.00295300

Dits

TS(Dits)

C	1.12805100	1.74105600	-0.27755300
H	1.52317300	1.31210400	-1.22914100
H	0.49681700	2.60360100	-0.58113400
H	1.99917500	2.17185100	0.24725200
C	0.47646700	0.77578800	0.65811500
H	0.83872200	0.38709600	1.60200700
H	1.73653500	-0.63861400	0.21803300
Br	-1.07214500	-0.23636300	-0.04742500
O	2.41999900	-1.33989200	-0.03327700
H	1.94356200	-1.94526100	-0.61428200

$HO^-(H_2O_w) + CH_3CH_2Br$ (MP2):

1R

$HO^-(H_2O_w)$

O	-1.25585200	0.08659800	-0.07417600
H	-1.59198200	-0.53918100	0.58507500
O	1.25097800	-0.09815100	-0.05013800
H	1.46955400	0.68188300	0.47539500
H	0.16141900	-0.05028400	-0.06595500

CH_3CH_2Br

C	-2.07597900	-0.40288300	-0.00000400
H	-1.98325600	-1.03768400	-0.89320200
H	-3.07803800	0.05833300	0.00093500
H	-1.98183100	-1.03909600	0.89213400
C	-1.01939700	0.69212600	0.00006500
H	-1.07574300	1.32389700	-0.89603500
H	-1.07525500	1.32333800	0.89625200
Br	0.79332500	-0.06755000	-0.00001300

anti-E2

1RC(aE)

C	0.41642400	1.45453600	0.80180100
H	-0.16844700	2.33731900	0.49681700
H	1.49534100	1.66044600	0.69023200
H	0.17479000	1.21105900	1.84850900
C	0.15158200	0.27407000	-0.11009000
H	0.39186300	0.49917400	-1.15397200
H	0.69605500	-0.62228600	0.20814400
Br	-1.78450800	-0.23117700	-0.10022600
O	2.93828200	0.72360200	-0.75698900
H	3.84646700	0.90083600	-1.04224100
O	2.90335300	-1.43058600	0.56199600
H	2.96910700	-0.51508500	0.00805300
H	2.91149700	-2.09602600	-0.13795800

1TS(aE)

C	0.60445100	0.50001000	0.72808100
H	0.36397500	1.48053900	1.16769500
H	1.82619400	0.61737900	0.15976800
H	0.69063700	-0.27786500	1.50116100
C	-0.22957800	0.13452000	-0.39856700
H	-0.33817800	0.90156300	-1.17413500
H	-0.02967900	-0.85253600	-0.83138900
Br	-2.36897400	-0.15917000	-0.00792900

O	3.00890000	0.71563200	-0.36805500
H	3.21751900	1.66175400	-0.36386800
O	5.16291100	-0.75619600	0.17592800
H	4.35039800	-0.18045800	0.02216800
H	5.20950700	-1.26207500	-0.64397600

1PC(aE)

C	2.06694500	-1.60567700	-0.03667000
H	0.97493700	-1.50840500	-0.07132200
H	1.48519000	0.42512900	1.51686800
H	2.49495300	-2.51711100	0.39545800
C	2.86568000	-0.62155900	-0.51055900
H	2.43261500	0.28851500	-0.93854100
H	3.95721400	-0.70939200	-0.47564900
Br	-1.54485500	-0.35064700	-0.08665100
O	0.82073400	1.08524000	1.76530400
H	0.00508800	0.68899300	1.37712600
O	0.71266900	2.09508600	-1.05158800
H	0.87720600	2.07576900	-0.09243200
H	-0.02024300	1.44994000	-1.10508700

1P1(E)

H_2O

O	0.00000000	0.00000000	0.11910300
H	0.00000000	0.76051500	-0.47641100
H	0.00000000	-0.76051500	-0.47641100

$CH_2=CH_2$

C	0.00000000	0.67466200	0.00000000
H	0.93365900	1.24294700	0.00000000
H	-0.93365200	1.24297000	0.00000000
C	0.00000000	-0.67466200	0.00000000
H	-0.93365900	-1.24294700	0.00000000
H	0.93365200	-1.24297000	0.00000000

syn-E2

1RC(sE)

C	0.50586200	-1.23672300	0.93384800
H	1.58283900	-1.43663600	0.79742400
H	-0.06400100	-2.17322300	0.82070100
H	0.31273400	-0.82258000	1.93619000
C	0.14377000	-0.23803800	-0.14657900
H	0.65931800	0.71934200	-0.01150000
H	0.33738100	-0.62848900	-1.15039100

Br	-1.81294300	0.18823200	-0.10735800
O	2.98231600	-0.79690500	-0.72218700
H	3.73799300	-0.81126900	-1.32758600
O	2.85487200	1.48765800	0.34036000
H	2.94608900	0.53070000	-0.13795300
H	3.34535100	1.35657400	1.16165800

1TS(sE)

C	0.33522000	1.83080400	0.15959200
H	0.90935700	2.32660500	-0.63826500
H	1.10237200	0.77543900	0.41736600
H	0.34271700	2.41292200	1.09373500
C	-1.02010100	1.46976900	-0.27789700
H	-1.86477800	1.86945600	0.29649000
H	-1.21394100	1.54074800	-1.35505900
Br	-1.52785000	-0.60006300	-0.03446500
O	1.88333000	-0.23809100	0.67513700
H	1.27895100	-0.98865100	0.56684400
O	4.23470600	-0.48912100	-0.50528300
H	3.30365500	-0.40968700	-0.11295600
H	4.78141300	-0.51036300	0.28912600

1PC(sE)

C	-2.86575900	-0.62146700	-0.51056100
H	-2.43273900	0.28868400	-0.93842600
H	-1.48532400	0.42505500	1.51668400
H	-3.95729200	-0.70929300	-0.47562400
C	-2.06697000	-1.60558900	-0.03676700
H	-2.49487400	-2.51706200	0.39525100
H	-0.97496400	-1.50821300	-0.07147100
Br	1.54490000	-0.35060600	-0.08664300
O	-0.82082600	1.08502200	1.76535200
H	-0.00524900	0.68894600	1.37705900
O	-0.71265300	2.09495300	-1.05155400
H	-0.87719200	2.07576900	-0.09241000
H	0.02032500	1.44986900	-1.10497500

inv-S_N2

1RC(iS)

C	0.58683800	-1.30358200	0.40259000
H	0.47306200	-1.13293500	1.48387100
H	1.66509300	-1.35526400	0.17189000
H	0.09425800	-2.25301900	0.13435500
C	0.02602300	-0.13093400	-0.37865200

H	0.54448800	0.80341600	-0.10587300
H	0.07367100	-0.29253400	-1.46377200
Br	-1.92054100	0.11456600	-0.01184200
O	2.41034100	1.42184700	0.28640500
H	2.89951100	2.24733400	0.15824000
O	3.86065500	-0.55055100	-0.34623700
H	4.34596800	-0.70243600	0.47465300
H	3.27775800	0.31236500	-0.10387300

1TS(iS)

C	0.64126700	-1.09522500	0.61146500
H	0.10860000	-1.37880600	1.53104000
H	1.72637500	-1.12635200	0.78192000
H	0.37273100	-1.81367600	-0.17619400
C	0.25884700	0.30521000	0.19562900
H	0.09093500	1.06647100	0.94639800
H	0.42396800	0.62475800	-0.82607500
Br	-2.02126500	0.02588700	-0.15153800
O	2.29695200	0.99404000	0.45413000
H	2.33199400	1.92261900	0.17468200
O	4.20512400	-0.39081800	-0.67431600
H	4.81906100	-0.46283400	0.06658900
H	3.45331100	0.17607800	-0.27560500

1PC(iS)

C	1.15988900	-0.99467800	0.43850900
H	1.60769500	-1.15496300	1.43299800
H	1.78283700	-1.49386100	-0.32216700
H	0.14718600	-1.42459900	0.41705200
C	1.03150200	0.48668800	0.14464100
H	0.35520700	0.96526500	0.86704300
H	0.61280500	0.64696100	-0.85972900
Br	-2.42085200	-0.02743400	-0.08952100
O	2.36835500	1.08471700	0.23875000
H	2.28300100	2.01461000	-0.00804100
O	4.69240600	-0.38440700	-0.42113500
H	4.41841400	-1.28620000	-0.21427500
H	3.88824200	0.13842500	-0.21946600

1P1(S)

CH_3CH_2OH

C	1.21675300	-0.24450000	0.02229700
H	2.09016800	0.42348600	-0.05359700
H	1.27271400	-0.96954700	-0.80679200

H	1.27352300	-0.79642800	0.97244200
C	-0.07613200	0.56147600	-0.04751200
H	-0.12781800	1.12975500	-0.99414600
H	-0.12678200	1.28407500	0.78005700
O	-1.24663600	-0.25899300	0.10850800
H	-1.25244100	-0.90125400	-0.61473800

ret-S_N2

1RC(rS)

C	-0.41662000	1.45358900	0.80299000
H	-0.17491800	1.20907400	1.84946700
H	-1.49558900	1.65939800	0.69166400
H	0.16806300	2.33685400	0.49888100
C	-0.15153800	0.27409100	-0.11012800
H	-0.69603700	-0.62260900	0.20712100
H	-0.39164000	0.50025300	-1.15381900
Br	1.78454300	-0.23111300	-0.10033800
O	-2.90385100	-1.42983800	0.56240300
H	-2.91306900	-2.09538900	-0.13736000
O	-2.93762800	0.72339700	-0.75822100
H	-2.96913800	-0.51458900	0.00801900
H	-3.84589300	0.90139900	-1.04275200

1TS(rS)

C	-0.91273600	1.94602300	0.29556500
H	-1.87634500	1.71093500	0.76159600
H	-0.97748700	2.89305000	-0.28099900
H	-0.16601600	2.09331800	1.09070700
C	-0.47010900	0.84733500	-0.63074700
H	-1.25094200	0.28621600	-1.14605100
H	0.28581900	1.20238900	-1.34020000
Br	1.63145500	-0.30126500	-0.03888000
O	-1.24258200	-0.52719700	0.84150900
H	-0.55754800	-1.21295300	0.81338100
O	-3.45504100	-0.92109800	-0.44625600
H	-2.56989700	-0.85249900	0.07044500
H	-4.11045500	-0.74995600	0.24098300

1PC(rS)

C	2.19963100	-0.49450000	1.10042100
H	2.77882100	0.44254000	1.12017500
H	2.71525300	-1.24450100	1.72695800
H	1.20153400	-0.29957800	1.52051100
C	2.06043500	-1.00809000	-0.33250500

H	3.05371100	-1.20829400	-0.77119700
H	1.48857900	-1.95422900	-0.33392700
Br	-1.54908900	-0.28546700	0.05733600
O	1.42392900	-0.05661800	-1.19349500
H	0.46078000	-0.10878800	-0.96492900
O	0.58268400	2.50166700	0.09592600
H	1.12505600	1.85224200	-0.38412500
H	-0.21891600	1.96709600	0.25284300

PT

1RC(PT)

C	0.41708600	1.45211200	0.80414000
H	-0.16725000	2.33579100	0.50058600
H	1.49610600	1.65761300	0.69281200
H	0.17535400	1.20705500	1.85047300
C	0.15147300	0.27332500	-0.10971900
H	0.39187600	0.50001100	-1.15322300
H	0.69543700	-0.62384400	0.20709700
Br	-1.78488200	-0.23086900	-0.10047100
O	2.93715500	0.72356200	-0.75943200
H	3.84564200	0.90250000	-1.04267300
O	2.90516500	-1.42889800	0.56248400
H	2.91439500	-2.09478600	-0.13705900
H	2.96941000	-0.51386600	0.00752300

1TS(PT)

C	-0.32322000	2.18807900	0.57076400
H	-0.31717500	2.55074400	-0.47203900
H	0.36010300	2.84366000	1.14656400
H	-1.34923400	2.33267800	0.97283800
C	0.23518000	0.76452000	0.66016500
H	1.69375700	0.82570300	-0.26858700
H	0.11694100	0.41974400	1.70928000
Br	-1.15065500	-0.43919300	-0.25599900
O	2.55793400	0.93692000	-0.84770200
H	2.51582100	0.16775800	-1.43229000
O	1.81821800	-1.98480200	0.69827000
H	0.89041500	-2.09060900	0.43587800
H	1.88131500	-1.01047200	0.67819300

1PC(PT)

C	0.19103000	2.07106200	0.43641600
H	0.38504900	2.28706900	-0.62908600
H	0.99329200	2.56518300	1.01958600

H	-0.77211300	2.55485200	0.70637200
C	0.27409700	0.56553500	0.72352600
H	1.94715900	0.54564900	-0.29739000
H	-0.02705200	0.41537400	1.78245500
Br	-1.33687900	-0.27224100	-0.22037300
O	2.79825300	0.57137000	-0.82074100
H	3.05766500	-0.35844000	-0.74453300
O	1.71294000	-1.93271200	0.59018700
H	1.01504300	-2.40522800	0.11660900
H	1.31141100	-1.00487000	0.64381300

1P1(PT)

CH_3CHBr^-

C	2.07549600	-0.36492300	0.02184600
H	2.05892300	-1.06978800	-0.82986600
H	3.09530600	0.07265900	0.05863700
H	1.93762000	-0.97265400	0.94941500
C	1.09888100	0.80214300	-0.19866200
H	1.10139100	1.38819400	0.75136100
Br	-0.77827200	-0.05833500	0.00375300

Dits

1TS(Dits)

C	0.57647000	1.71259200	0.01683900
H	1.16332200	1.19022100	-0.78485400
H	0.06732900	2.55806900	-0.49800000
H	1.31307000	2.16576300	0.70952300
C	-0.33657700	0.84328700	0.82358400
H	-0.17495500	0.48854300	1.83960100
H	0.92114700	-0.57981200	0.72586600
Br	-1.59507600	-0.22972400	-0.19704400
O	1.57414500	-1.34102900	0.54008800
H	1.08379100	-1.85074800	-0.11917400
O	3.86034700	-0.08810600	-0.44428000
H	3.45271500	0.75076600	-0.69611400
H	3.08592100	-0.58465100	-0.08930100
