# Supplementary material for: How Microsolvation Affects the Balance of Atomic Level Mechanism in Substitution and Elimination Reactions: Insights into the Role of Solvent Molecules in Inducing Mechanistic Transitions
Source: Molecules. 2025 Jan 23;30(3):496. doi: 10.3390/molecules30030496 (PMC11819716; doi:10.3390/molecules30030496)
Supplement: Supplementary file 1 [file molecules-30-00496-s001.zip › Supplementary Materials.pdf]

How Microsolvation Affects the Balance of Atomic Level Mechanism in Substitution and Elimination Reactions: Insight into the Role Solvent Molecules Plays in Inducing Mechanistic Transitions

Gang Fu [1], Hongyi Wang [1], Wenqing Zhen [1], Xin Zhou [1], Li Yang [2,*] and Jiaxu Zhang [1,2,*]

[1] State Key Laboratory of Urban Water Resource and Environment, School of Chemistry and Chemical Engineering, MIIT Key Laboratory of Critical Materials Technology for New Energy Conversion and Storage, Harbin Institute of Technology, Harbin 150001, P. R. China

[2] Key Laboratory of Chemistry and Chemical Engineering on Heavy-Carbon Resources, School of Chemistry and Chemical Engineering, Yili Normal University, Yining 835000, P. R. China

*Author E-mail Address: yangli_ylnu@163.com and zhjx@hit.edu.cn

Table of Contents

Tables S1-S2: Pages S3

Figure S1-S15: Pages S4-S12

Animations S1–S8: Pages S13

Table S1. Energy decomposition analysis (EDA) of the anti-E2 and inv-S_N2 transition states of the $HO^{-}(H_2O_w)_{n=0-3}$ + CH_3CH_2Br reactions, Energy unit, kcal mol^{-1}.

Species	ΔE_1	ΔE_2	ΔE_{strain}	ΔE_{int}	ΔE_{TS}
$0H_2O_w$-aE	0.01	3.81	3.82	-23.62	-19.8
$1H_2O_w$-aE	1.37	17.40	18.77	-30.47	-11.7
$2H_2O_w$-aE	2.30	26.07	28.37	-33.08	-4.71
$3H_2O_w$-aE	4.97	34.77	39.74	-35.49	4.25
$0H_2O_w$-iS	0.00	4.71	4.71	-25.13	-20.42
$1H_2O_w$-iS	0.75	12.23	12.98	-26.22	-13.24
$2H_2O_w$-iS	0.77	17.26	18.03	-26.15	-8.12
$3H_2O_w$-iS	2.66	22.66	25.32	-26.19	-0.87

Note: Here ΔE_1 is the strain energy change of the $HO^{-}(H_2O_w)_{n=0-3}$ from the reactant to the transition state, ΔE_2 is the strain energy change of CH_3CH_2Br from the reactant to the transition state, $\Delta E_{strain} = \Delta E_1 + \Delta E_2$ and $\Delta E_{TS} = \Delta E_{strain} + \Delta E_{int}$, Energy unit, kcal mol^{-1}.

Table S2. Energy decomposition analysis (EDA) of the interaction energies of the anti-E2 and inv-S_N2 transition states of the $HO^{-}(H_2O_w)_{n=0-3}$ + CH_3CH_2Br reactions, Energy unit, kcal mol^{-1}.

Species	E_{els}	E_{orb}	E_x	E_{rep}	E_{DFTc}	E_{dc}	E_{xrep}	E_c	E_{int}
$0H_2O_w$-aE	-30.73	-26.61	-21.83	62.10	-5.43	-1.12	40.27	-6.55	-23.62
$1H_2O_w$-aE	-51.86	-56.63	-42.74	129.35	-7.16	-1.43	86.61	-8.59	-30.47
$2H_2O_w$-aE	-60.04	-70.34	-50.61	157.81	-7.90	-2.00	107.20	-9.90	-33.08
$3H_2O_w$-aE	-66.04	-79.91	-55.55	176.49	-8.26	-2.22	120.94	-10.48	-35.49
$0H_2O_w$-iS	-30.80	-22.50	-19.29	55.03	-6.13	-1.44	35.74	-7.57	-25.13
$1H_2O_w$-iS	-37.02	-28.54	-24.13	72.77	-7.04	-2.26	48.64	-9.30	-26.22
$2H_2O_w$-iS	-39.98	-32.07	-26.95	84.07	-7.85	-3.37	57.12	-11.22	-26.15
$3H_2O_w$-iS	-42.97	-35.94	-29.92	95.29	-8.60	-4.05	65.37	-12.65	-26.19

Note: $\Delta E_{int} = \Delta E_{els} + \Delta E_{xrep} + \Delta E_{orb} + \Delta E_c$, $\Delta E_{xrep} = \Delta E_x + \Delta E_{rep}$, $\Delta E_c = \Delta E_{DFTc} + \Delta E_{dc}$. where ΔE_{int}: interaction energy, ΔE_{els}: electrostatic energy, ΔE_x: exchange energy, ΔE_{rep}: Pauli repulsion, ΔE_{orb}: orbital interaction, ΔE_{DFTc}: correlation energy, ΔE_{dc}: dispersion correction.

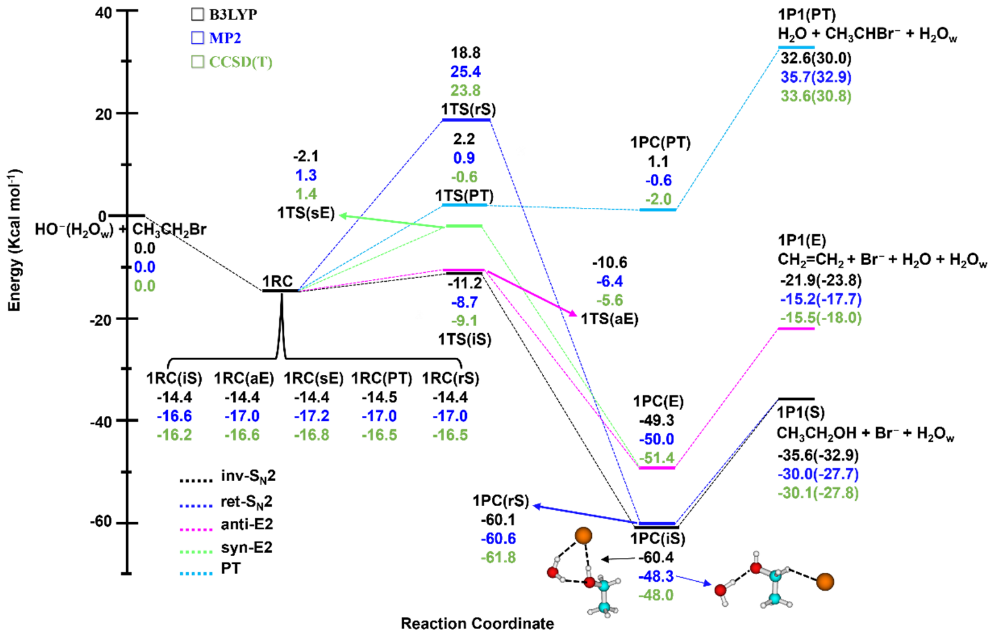

Figure S1. The classical relative energies of HO$^-$(H_2O_W) + CH_3CH_2Br reactions under B3LYP/ECP/d, MP2/ECP/d and CCSD(T)/PP/t//MP2/ECP/d methods. The reported energies in kcal mol^{-1} are relative to the isolated molecules and ions without zero-point energy (ZPE). Numbers in parentheses include ZPE.

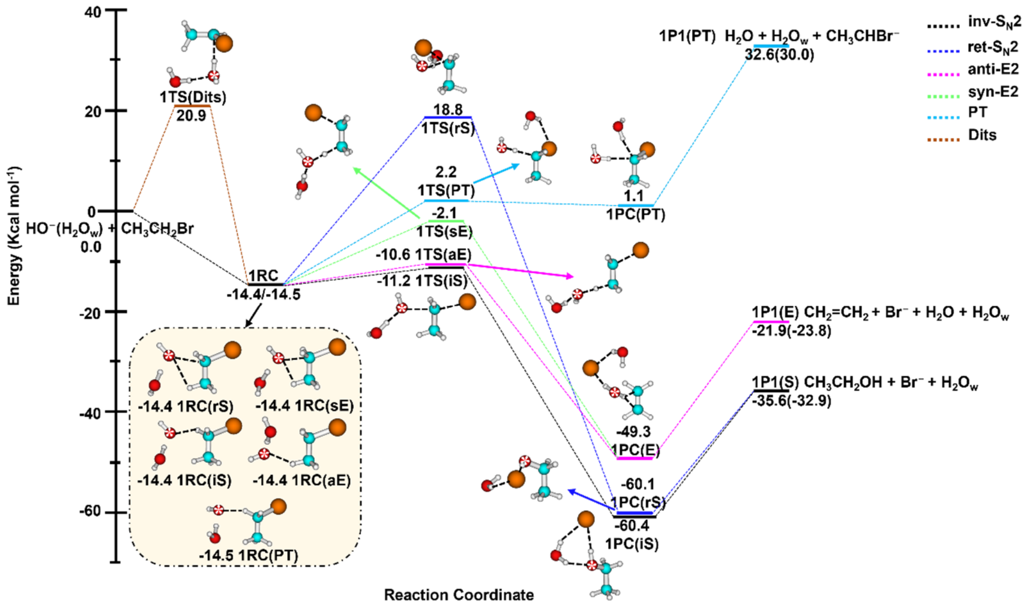

Figure S2. Potential energy profile and stationary points for S_N2, E2, and PT pathways in reaction HO$^-$(H_2O_W) + CH_3CH_2Br at the B3LYP/ECP/d level of theory. Energies in kcal mol^{-1} are relative to reactants without zero-point energy (ZPE). The numbers in parentheses denote calculated reaction energies with ZPE included. Note that here oxygen atoms with asterisks represent O and oxygen atoms without asterisks represent O_W.

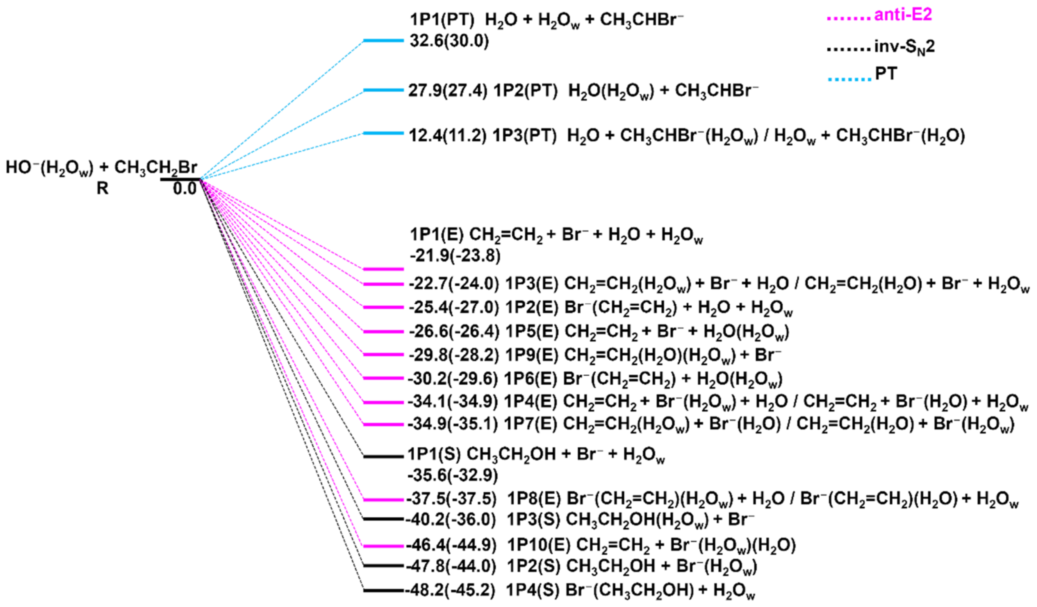

Figure S3. Classical relative energies of the isolated and complexed products of the S$_N$2, E2, and PT pathways in the $HO^-(H_2O_w) + CH_3CH_2Br$ reaction at the B3LYP/ECP/d level. Energies in kcal mol^{-1} are relative to reactants without zero point energy (ZPE). The numbers in parentheses denote calculated reaction energies with ZPE included.

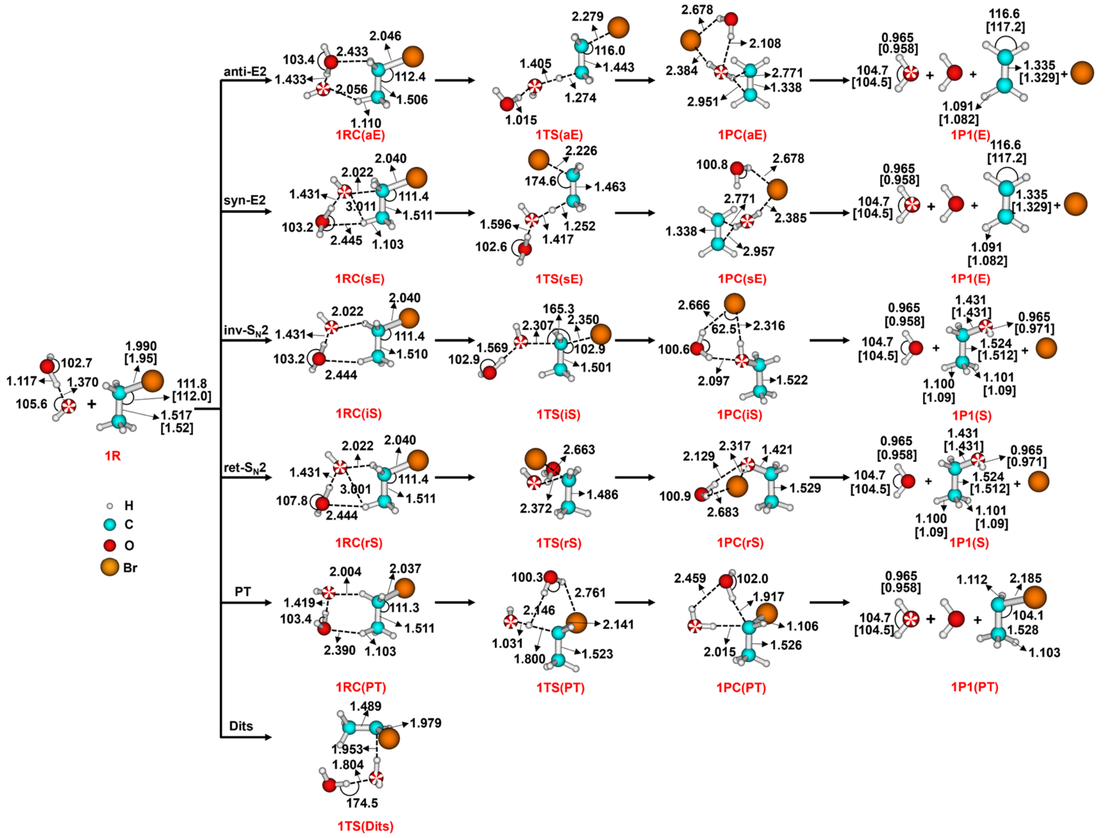

Figure S4. Stationary point structures of S$_N$2, E2 and PT channels in the $HO^-(H_2O_w) + CH_3CH_2Br$ reaction optimized at the B3LYP/ECP/d level of theory. Bond distances (in

Å) and angles (in degree) are shown for each reaction pathway and the available experimental data [a,b] are given in parentheses. [a] Lide, D. R. CRC Handbook of Chemistry and Physics; CRC Press: Boca Raton, FL, 2005. [b] Chase, M. W., Jr.; Davies, C. A.; Downey, J. R., Jr.; Frurip, D. J.; McDonald, R. A.; Syverud, A. N. JANAF Thermochemical Tables; National Bureau of Standards: Washington, D.C., 1985; Vol. 14.

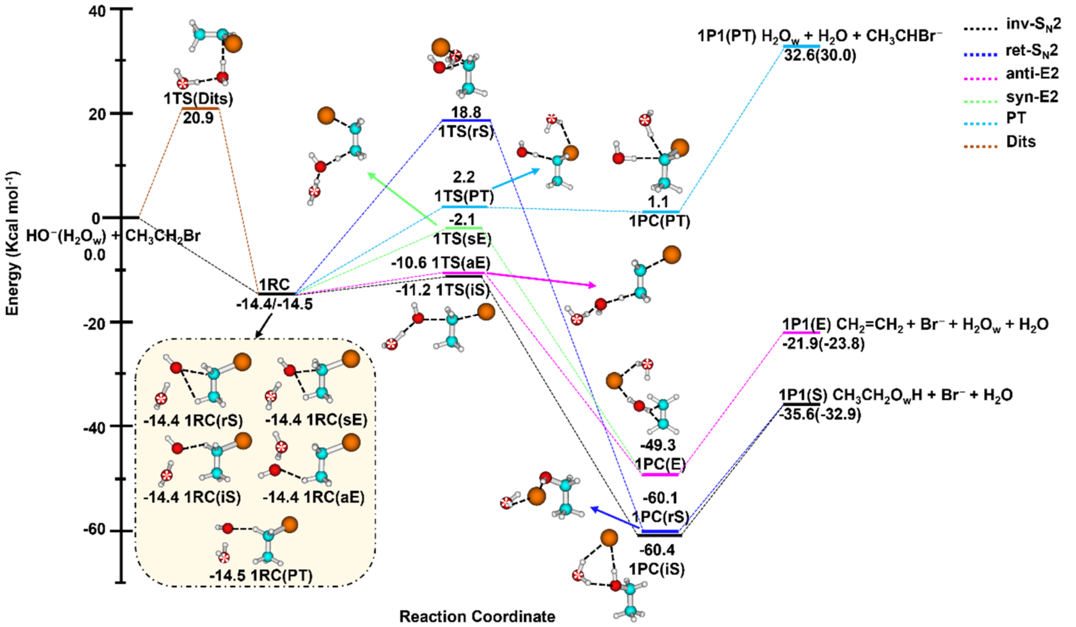

Figure S5. Potential energy profile and stationary points for O_W-induced S_N2, E2 and PT pathways in reaction $HO^-(H_2O_W) + CH_3CH_2Br$ at the B3LYP/ECP/d level of theory. Energies in kcal mol^{-1} are relative to reactants without zero-point energy (ZPE). The numbers in parentheses denote calculated reaction energies with ZPE included. Note that here oxygen atoms with asterisks represents O and oxygen atoms without asterisks represent O_W.

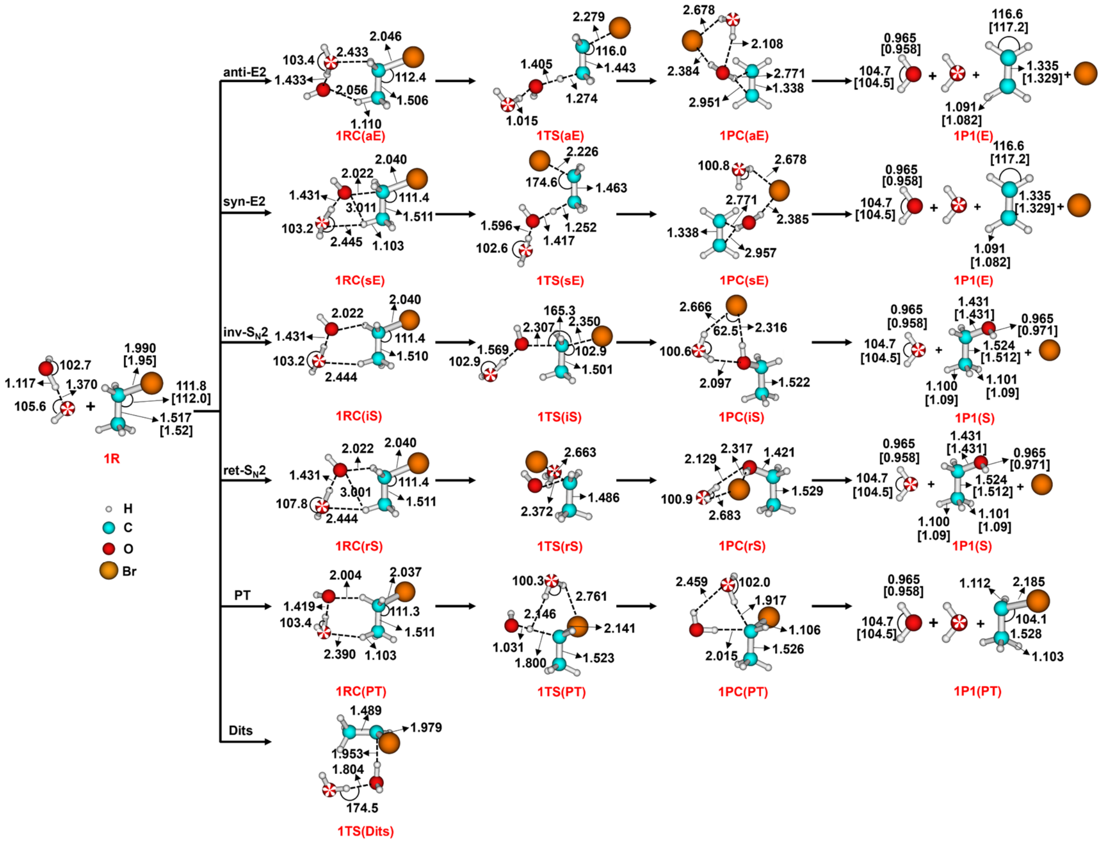

Figure S6. Stationary point structures of O_w-induced S_N2, E2, and PT channels in the $HO^{-}(H_2O_w) + CH_3CH_2Br$ reaction optimized at the B3LYP/ECP/d level of theory. Bond distances (in Å) and angles (in degree) are shown for each reaction pathway and the available experimental data (refs a and b as in Figure. S4) are given in parentheses.

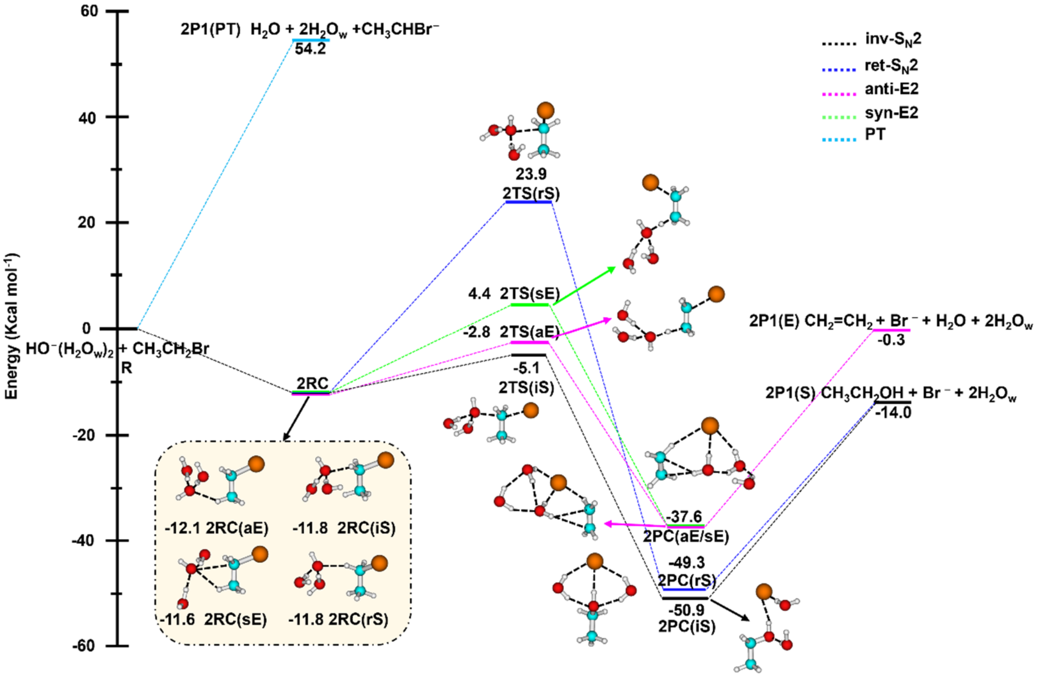

Figure S7. Potential energy profile and stationary points for S_N2, E2, and PT pathways in reaction $HO^-(H_2O_w)_2 + CH_3CH_2Br$ at the B3LYP/ECP/d level of theory. Energies in kcal mol^{-1} are relative to reactants without zero-point energy (ZPE). The numbers in parentheses denote calculated reaction energies with ZPE included.

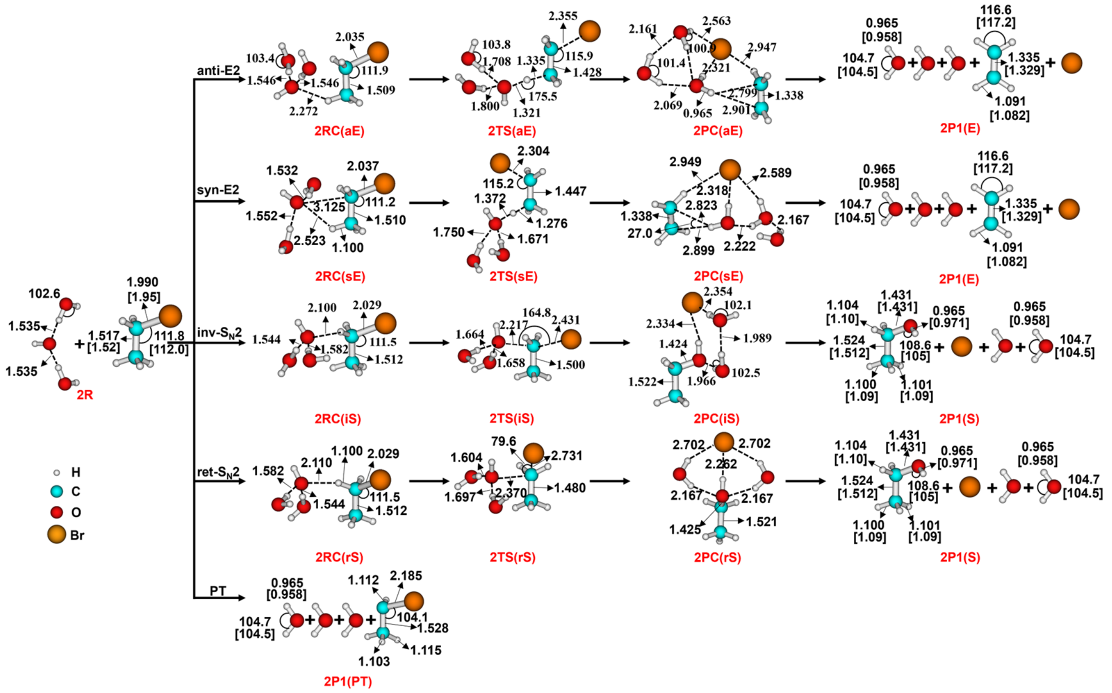

Figure S8. Stationary point structures of S_N2, E2, and PT channels in the $HO^-(H_2O_w)_2$ + CH_3CH_2Br reaction optimized at the B3LYP/ECP/d level of theory. Bond distances (in Å) and angles (in degree) are shown for each reaction pathway and the available experimental data (refs a and b as in Figure S4) are given in parentheses.

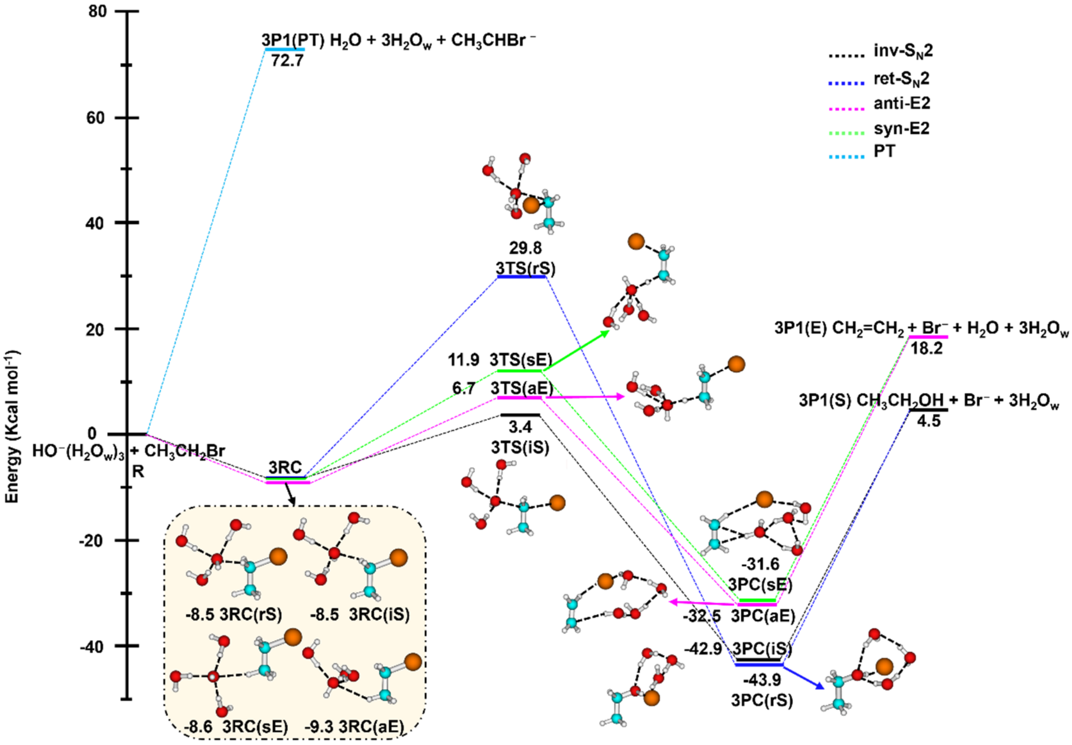

Figure S9. Potential energy profile and stationary points for S_N2, E2, and PT pathways in reaction $HO^-(H_2O_W)_3 + CH_3CH_2Br$ at the B3LYP/ECP/d level of theory. Energies in kcal mol^{-1} are relative to reactants without zero-point energy (ZPE). The numbers in parentheses denote calculated reaction energies with ZPE included.

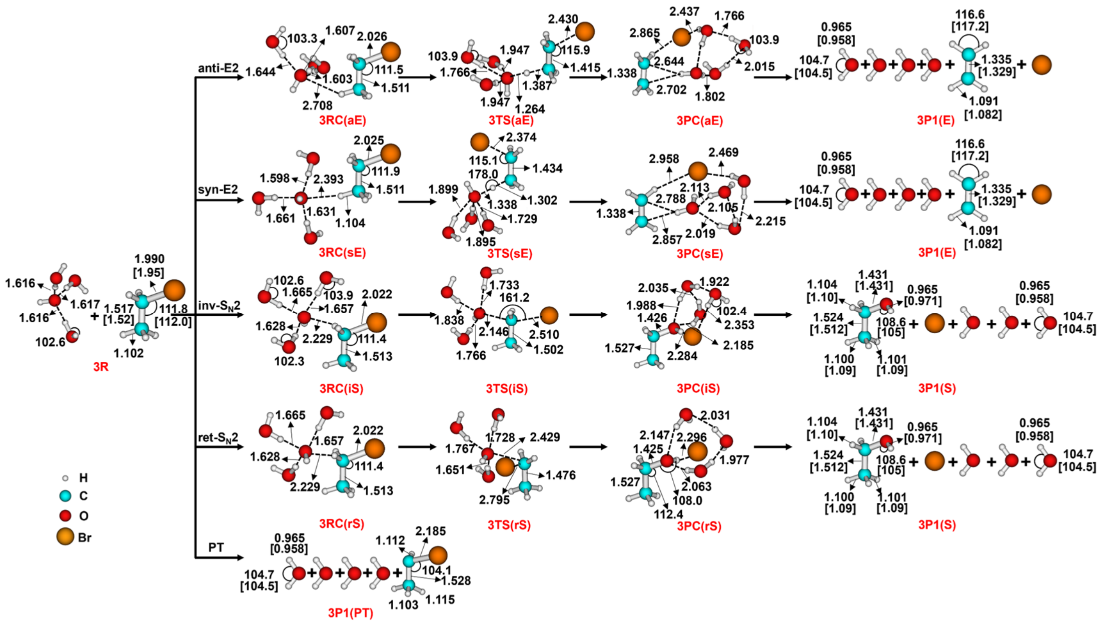

Figure S10. Stationary point structures of S_N2, E2, and PT channels in the $HO^-(H_2O_W)_3$

+ CH3CH2Br reaction optimized at the B3LYP/ECP/d level of theory. Bond distances (in Å) and angles (in degree) are shown for each reaction pathway and the available experimental data (refs a and b as in Figure S4) are given in parentheses.

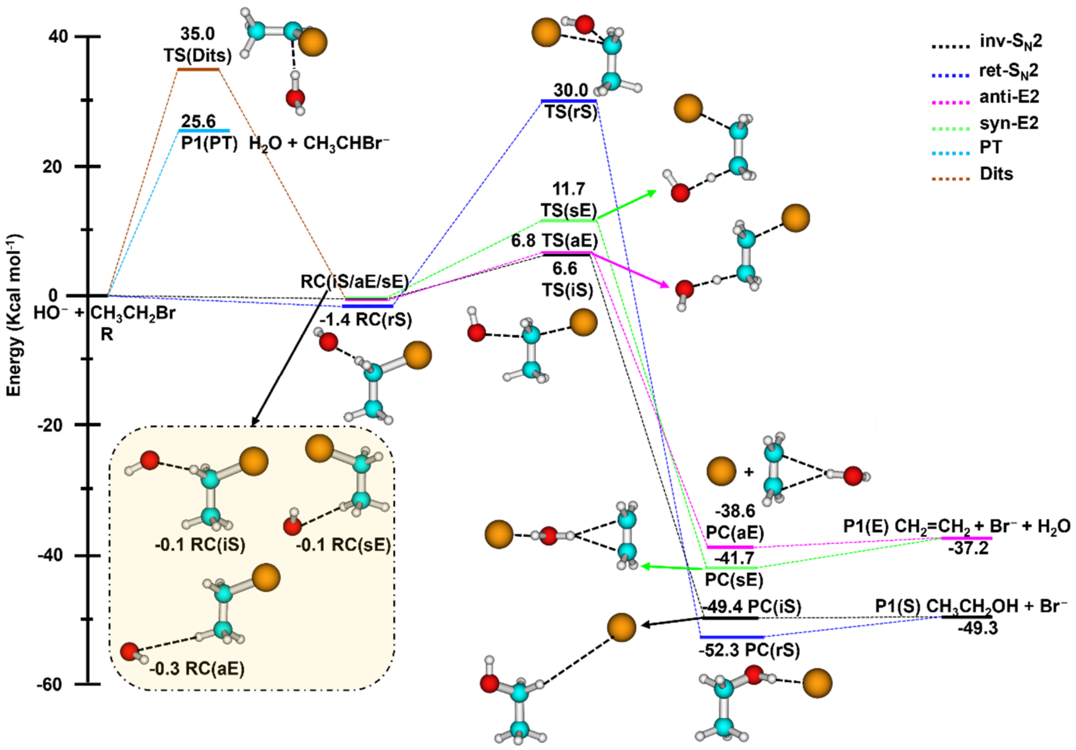

Figure S11. Potential energy profile and stationary points for S_N2, E2, and PT pathways in reaction HO^-(PCM, water) + CH_3CH_2Br at the B3LYP/ECP/d level of theory. Energies in kcal mol^{-1} are relative to reactants without zero-point energy (ZPE). The numbers in parentheses denote calculated reaction energies with ZPE included.

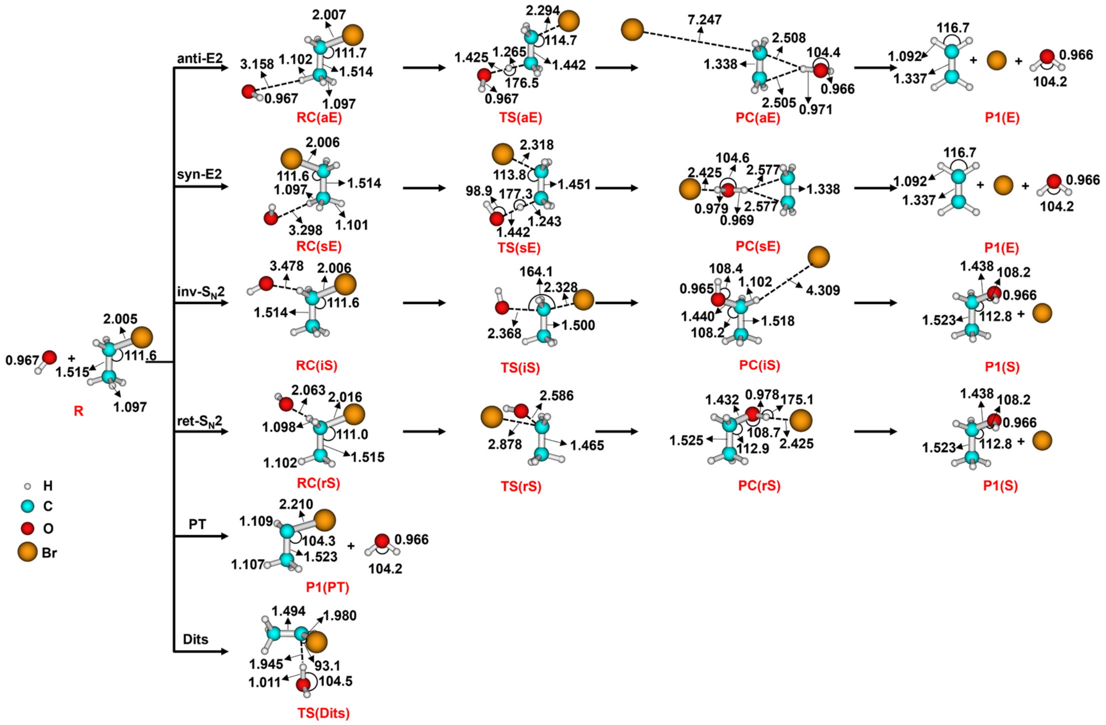

Figure S12. Stationary point structures of S$_N$2, E2, and PT channels in the HO$^-$(PCM, water) + CH_3CH_2Br reaction optimized at the B3LYP/ECP/d level of theory. Bond distances (in Å) and angles (in degree) are shown for each reaction pathway and the available experimental data (refs a and b as in Figure. S4) are given in parentheses.

Species	$0H_2O_w$	$1H_2O_w$	$2H_2O_w$	$3H_2O_w$
aE	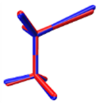 0.07	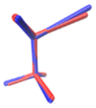 0.13	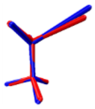 0.16	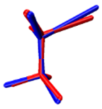 0.19
iS	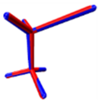 0.05	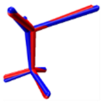 0.11	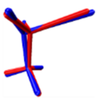 0.14	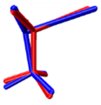 0.17

Figure S13. Root mean square deviation (RMSD) of CH_3CH_2Br fragment from initial reactant CH_3CH_2Br in the transition state structure of inv-S$_N$2 and anti-E2 in HO$^-$ $(H_2O_W)_{n=0-3}$ + CH_3CH_2Br reaction. Red and blue represent the CH_3CH_2Br segments in the reactants and transition states, respectively. RMSD is defined as follows:

$$\text{RMSD} = \sqrt{\frac{1}{N}\sum_i^{natom}[(x_i^{TS}-x_i^R)^2+(y_i^{TS}-y_i^R)^2+(z_i^{TS}-z_i^R)^2]}$$

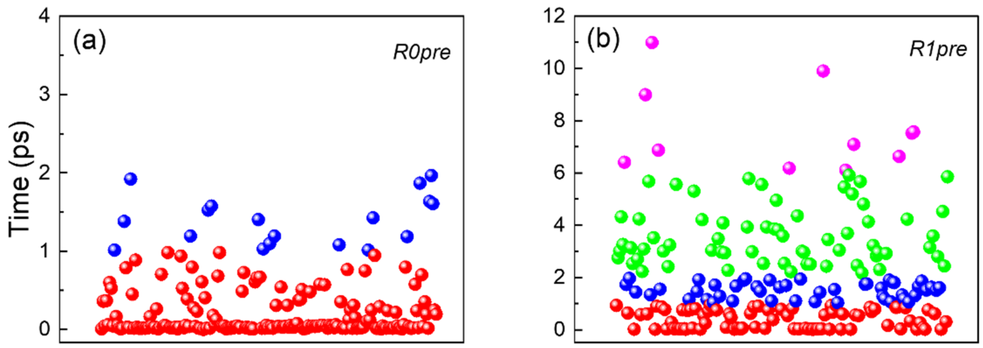

Figure S14. Interaction time, defined as the time between the first point of collision and the onset of the transition state. (a) Interaction time for the pre mechanism in HO^- + CH_3CH_2Br (b) Interaction time for the pre-mechanism in $HO^-(H_2O_w)$ + CH_3CH_2Br.

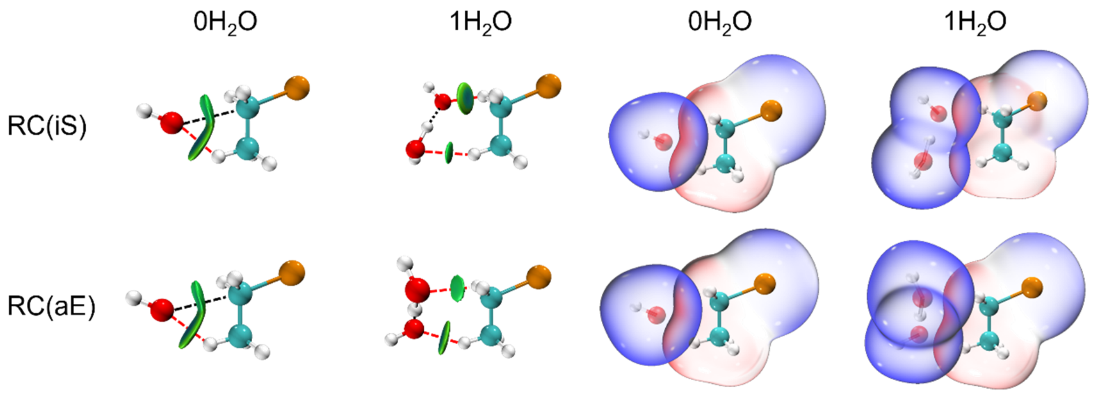

Figure S15. IGMH (left), ESP (right) analysis of $HO^-(H_2O_w)_{n=0\text{-}1}$ + CH_3CH_2Br reaction entrance channel complexes

Animations S1 – S8

Animation S1. A trajectory showing the indirect mechanism that proceeds via 1RC complexes in the entrance channel (see Figure. S2) for the solvated O-anti-E2 reaction $HO^{-}(H_2O_w) + CH_3CH_2Br \rightarrow HO\text{-}H + CH_2{=}CH_2 + Br^{-} + H_2O_w$.

Animation S2. A trajectory showing the indirect mechanism that proceeds via 1RC complexes in the entrance channel (see Figure. S5) for the solvated O_w-anti-E2 reaction $HO^{-}(H_2O_w) + CH_3CH_2Br \rightarrow HO_w\text{-}H + CH_2{=}CH_2 + Br^{-} + H_2O$.

Animation S3. An indirect trajectory occurs by the formation of 1RC complexes for solvated O-inv-S_N2 reaction $HO^{-}(H_2O_w) + CH_3CH_2Br \rightarrow CH_3CH_2OH + Br^{-} + H_2O_w$.

Animation S4. An indirect trajectory occurs by the formation of 1RC complexes for solvated O_w-inv-S_N2 reaction $HO^{-}(H_2O_w) + CH_3CH_2Br \rightarrow CH_3CH_2O_wH + Br^{-} + H_2O$.

Animation S5. An direct rebound trajectory occurring for solvated anti-E2 reaction $HO^{-}(H_2O) + CH_3CH_2Br \rightarrow CH_2{=}CH_2 + Br^{-} + 2H_2O$.

Animation S6. An direct strpping trajectory occurring for solvated anti-E2 reaction $HO^{-}(H_2O) + CH_3CH_2Br \rightarrow CH_2{=}CH_2 + Br^{-} + 2H_2O$.

Animation S7. A direct rebound trajectory occurs for solvated inv-S_N2 reaction $HO^{-}(H_2O) + CH_3CH_2Br \rightarrow CH_3CH_2OH + Br^{-} + H_2O$.

Animation S8. A direct stripping trajectory occurs for solvated inv-S_N2 reaction $HO^{-}(H_2O) + CH_3CH_2Br \rightarrow CH_3CH_2OH + Br^{-} + H_2O$.

Notes: In the animation, the color coding for atoms is as follows: C_α, C_β, H, H in hydroxide ion, O in hydroxide ion, O in water molecules, and Br for blue, cyan, white, silver, magenta, red and pink, respectively.
